# Self-supervised learning on graphs predicts non-coding RNA and disease associations

**DOI:** 10.1038/s41598-026-36030-2

**Published:** 2026-01-14

**Authors:** Qingwen Wu, Sujuan Tang

**Affiliations:** 1 Department of Data Center, Affiliated Hospital of Jining Medical University, Jining, China; 2 Department of Neurological Care Unit, Affiliated Hospital of Jining Medical University, Jining, China

**Keywords:** Non-coding RNA-disease, Association prediction, Graph neural network, Self-Supervised learning, Contrastive learning, Cancer, Computational biology and bioinformatics, Mathematics and computing

## Abstract

Non-coding RNAs (ncRNAs) play crucial roles in regulating the initiation and progression of various cancers. Accurate identification disease-related ncRNAs would provide a unique opportunity to design better therapeutic interventions. Graph convolutional network-based methods have been proposed to identify potential ncRNA-disease associations (RDAs). However, some methods only use the graph structure and ignore the similarity information of nodes, and some methods integrate multi-source relation data which will introduce noise and have poor generalization. Learning robust node embeddings using graph convolutional network to build RDA predictive frameworks with high generalization remains a key challenge. We proposed a new RDA prediction scheme, SSLGRDA, composed of graph self-supervised learning and machine learning. Since SSLGRDA works on both heterogeneous and homogeneous graphs, we constructed ncRNA-disease heterogeneous graph based on known RDA, as well as heterogeneous graph based on known RDA, ncRNA similarities and disease similarities. For the latter, we ignored the node and edge types and treated it as homogeneous graph. Based on multiple contrastive or generate strategies, we used graph self-supervised learning to extract robust ncRNA and disease embedding to enhance the prediction ability and generalization of the model. Finally, we use machine learning methods to predict latent RDA probabilities. To evaluate model performance, we performed SSLGRDA on 9 ncRNA-disease datasets. Comprehensive experimental results show that SSLGRDA not only has good generalization, but also outperforms several state-of-the-art methods. Case studies on three ncRNA-disease datasets further demonstrate the ability of SSLGRDA in discovering potential ncRNA-disease associations.

**Supplementary Information:**

The online version contains supplementary material available at 10.1038/s41598-026-36030-2.

## Introduction

As a template for protein synthesis, messenger RNAs (mRNAs) have become the major research focus for a long time, while non-coding RNAs (ncRNAs) were considered as by-products of massive transcription with less biological meaning^1^. Pervasive transcription produces a vast repertoire of ncRNAs of all sizes and shapes, including microRNAs (miRNAs), long non-coding RNAs (lncRNAs), and circular RNAs (circRNAs)^2^. Work from the past decade has altered our perception of ncRNAs from ‘junk’ transcriptional products to functional regulatory molecules that mediate cellular processes including chromatin remodeling, transcription, post-transcriptional modifications and signal transduction^3^. Most ncRNAs are now known as key regulators in various networks in which they could lead to specific cellular responses and fates. Especially in major malignant diseases, ncRNAs have been identified as oncogenic drivers and tumor suppressors^4^.

Clinicopathological findings and studies have elucidated the multi-faceted and frequently divergent effects ncRNAs impose both directly and indirectly on the formation and progression of complex disease. Potential new therapeutic targets and strategies have been identified from these findings, which may pave the way for further translational research and potentially advance clinical applications. Traditional experimental methods remain the most reliable approach for identify ncRNA-disease associations (RDAs), but the process is complex and time-consuming. Computational tools can augment existing knowledge, guide biological and biomedical applications and reduce costly experimental efforts. Widely used strategies for computationally predicting ncRNA-disease associations can be broadly categorized into three main classes: matrix transformation (MT), machine learning (ML), and graph neural network (GNN)-based methods.

MT strategy mainly uses matrix factorization algorithm. MFLDA^5^ decomposed lncRNA-disease heterogeneous matrix into low-rank matrices by matrix tri-factorization and optimized, and then the association matrix was reconstructed using the optimized low-rank matrices. MRLDC^6^ developed a matrix factorization with dual manifold regularization to infer potential circRNA-disease associations. However, those methods are shallow learning ones which cannot fully extract deep and complex associations between ncRNAs and diseases. DBN-MF^7^ applied deep belief network-based matrix factorization to predict miRNA-disease association. DBN-MF only rely on the confirmed real existing associations, but ignores ncRNA and disease attribute information. CKA-HGRTMF^8^ proposed a ncRNA-disease association prediction method of three-matrix factorization with hypergraph regularization terms (HGRTMF) based on central kernel alignment (CKA). Nevertheless, CKA-HGRTMF requires multiple ncRNA/disease similarity information to improve model performance. RNMFLP^9^ combined robust nonnegative matrix factorization and label propagation algorithm to predict circRNA-disease associations. These methods rely on the definition of similarity. At present, it is difficult to have an evaluation method to illustrate the accuracy of the definition of similarity. Therefore, the matrix-based method has the problem of how to verify the rationality of the definition of similarity, and the balance between avoiding noise and introducing more evidence should be carefully taken into consideration.

ML strategy mainly uses traditional machine learning and convolutional neural network (CNN) algorithms. MDA-CNN^10^ employed an autoencoder to learn the essential features of each miRNA-disease pair and utilized a convolutional neural network to predict the final label. RFLDA^11^ implemented a random forest and feature selection based lncRNA-disease prediction model. CDASOR^12^ proposed a method for predicting circRNA-disease associations based on convolutional and recurrent neural networks. Deepthi et al.^13^ introduced an ensemble method (AE-RF) by combining a deep autoencoder and a random forest to predict circRNA-disease association. Although CNN can extract latent features effectively, it can only learn local interactive features, but not global features. Additionally, most of the above methods ignore the topological information of ncRNA-disease network, and unable to learn the high order relations between them.

However, standard ML and CNN-based methods often treat associations as independent samples, largely ignoring the complex topological information of the ncRNA-disease network and the high-order relations between nodes. Recently, due to the strong modeling ability of graph convolutional network (GCN) on graph structure data, and the biochemical relation-ship between ncRNA and disease can be regarded as graph structure, GCN algorithm has gradually been used to predict unknown RDAs. GATMDA^14^ proposed a novel attention-based framework to integrate multi-source information effectively. Similarly, GGAECDA^15^ explored high-order graph correlations to enhance prediction accuracy, while NAGTLDA^16^ introduced a robust learning strategy to mitigate data noise and sparsity issues. These works highlight the growing importance of learning robust representations from complex graph structures. LR-GNN^17^ presented a graph neural network based on link representation to identify potential ncRNA-disease associations. LR-GNN applied a GCN-encoder to obtain node embedding and designed a propagation rule that captures the node embedding to construct the link representation. However, it ignored the semantic feature of the RDA network. GMNN2CD^18^ employed a graph Markov neural network algorithm to predict unknown circRNA-disease associations. MINIMDA^19^ fused mixed high-order neighborhood information of miRNAs and diseases in multimodal networks via GCN, and feed them into the multilayer perceptron to predict miRNA-disease underlying associations. Nevertheless, integrating different types of data effectively is a challenging task. Considering the heterogeneity of RDA networks, researchers introduced multiple association data to construct heterogeneous graphs, and used heterogeneous GCNs to identify unknown RDAs. However, reliable labeling data is expensive and difficult to obtain, and fusing multi-source data may bring the noise to the models. Overall, although GCN models have achieved remarkable success in the task of ncRNA-disease association prediction, they rely on a large amount of annotated association data on the one hand and rich feature transformation operations on the other. This leads to the disadvantages of severe label dependence, poor generalization and over-parameterized. Therefore, it is still a challenge to build GCN models with high generalization ability based on RDA network that can learn more effective representations from limited labeled data.

**Fig. 1:**
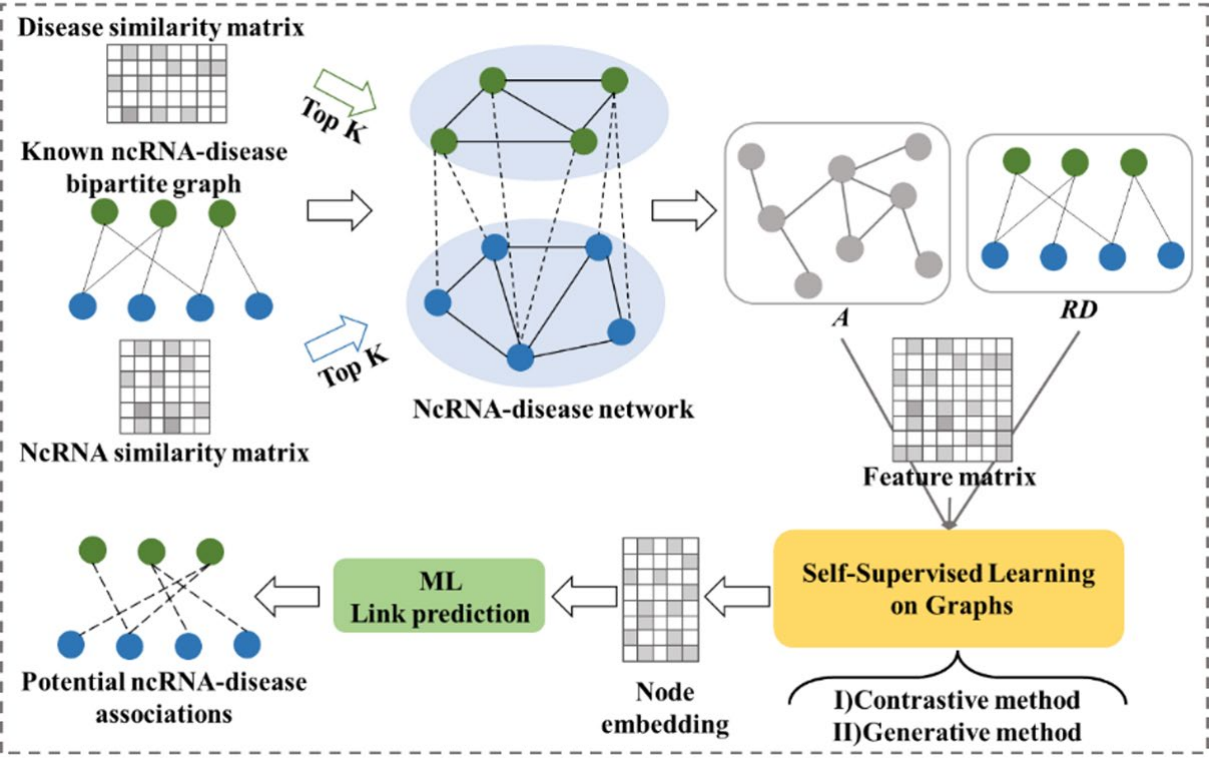
The flowchart of SSLGRDA. Mining robust node representation using graph self-supervised learning. Based on different self-supervised learning modes and input structures, SSLGRDA is further divided into six sub-models. See Materials and methods for details.

Inspired by the significance of graph self-supervised learning methods, we construct a Graph Self-Supervised Learning (SSL) Prediction scheme and named SSLGRDA. The flowchart of SSLGRDA is shown in Fig. 1. Simply put, given an ncRNA disease association graph and node attribute features, we obtain node embedding using SSL contrastive method or generative method, and then feed them into ML for link prediction. This framework provides a new simple and effective solution for solving homogeneous or heterogeneous graph-based RDA prediction problems.

## Materials and methods

In order to comprehensively evaluate model generalization, we collected multiple ncRNA-disease datasets from different literatures, which are widely used for RDA prediction. Specifically, Human circRNA-disease association (CDA) dataset was downloaded from CircR2Disease^20^. CircRNA similarity and disease similarity was downloaded from CKA-HGRTMF and GMNN2CD, respectively. In addition, we constructed another circRNA-disease association dataset based on^21^. Human lncRNA-disease association (LDA) dataset was downloaded from LncRNADisease^22^ and MNDR^23^. LncRNA similarity and disease similarity were downloaded from MFLDA, IDSSIM^24^ and MCGLDA^25^, respectively. Human miRNA-disease association (MDA) datasets were downloaded from HMDD2.0^26^ and HMDD3.0^27^, respectively. MiRNA similarity and disease similarity were downloaded from CKA-HGRTMF and MINIMDA, respectively.

Table 1 shows the statistical information of each ncRNA-disease dataset. CDA1 contains 585 circRNAs and 88 diseases, and 650 CDAs. CDA2 contains 590 circRNAs and 88 diseases, and 650 CDAs. CDA3 contains 533 circRNAs and 89 diseases, and 595 CDAs. LDA1 contains 240 lncRNAs and 386 diseases, and 2093 LDAs. LDA2 contains 89 lncRNAs and 190 diseases, and 1529 LDAs. LDA3 contains 194 lncRNAs and 128 diseases, and 577 LDAs. MDA1 and MDA2 contain 495 miRNAs and 383 diseases, and 5430 MDAs. MDA3 contains 788 miRNAs and 374 diseases, and 8968 MDAs. It is worth noting that the above ncRNA-disease datasets differ by the ncRNA (and disease) similarities, as each work has their own approach to processing.

**Table 1:** The statistical information of ncRNA-disease datasets.

Dataset	NcRNAs	Diseases	Links	Sparseness	Source
CDA1	585	88	650	0.0126	PMID:33,443,536
CDA2	590	88	651	0.0125	PMID: 32,241,268
CDA3	533	89	595	0.0125	PMID: 35,157,027
LDA1	240	386	2093	0.0225	PMID:29,228,285
LDA2	89	190	1529	0.0904	PMID:32,736,513
LDA3	194	128	577	0.0232	PMID:32,153,646
MDA1	495	383	5430	0.0286	PMID:35,524,503
MDA2	495	383	5430	0.0286	PMID:33,443,536
MDA3	788	374	8968	0.0304	PMID:35,524,503

### NcRNA-disease heterogeneous graph

Given *r* ncRNAs and *d* diseases, they are regarded as two types of vertices, and the associations between them are regarded as edges, which constitutes a heterogeneous graph of ncRNAs and diseases. The adjacency matrix is denoted by $RD \in {\mathbb{R}}^{r \times d}$. If there is a known association between ncRNA *i* and disease *j*, $RD_{i,j} = 1$, otherwise $RD_{i,j} = 0$.

### NcRNA-disease homogeneous graph

Furthermore, we ignore types of nodes and edges, and combine *RD*, ncRNA similarity and disease similarity to construct ncRNA-disease homogeneous graph. Specifically, the ncRNA similarity is represented by *SR* ∈ ${\mathbb{R}}$ ^*r*×*r*^, and the disease similarity is denoted by *SD* ∈ ${\mathbb{R}}$ ^*d*×*d*^. We only consider the top *k* (set to 5 based on our previous work GAERF) most similar lncRNAs (or diseases) for each row in the *SR* (or *SD*), and set them values to 1, the other values in the same row to 0. This threshold was selected to ensure that each node has sufficient neighbors for effective feature aggregation while avoiding the introduction of noise from weakly correlated nodes. After preprocessing, we obtain new similarity matrix *SR* (or *SD*). Then, we spliced *SR*, *SD* and *RD* together to form an ncRNA-disease homogeneous graph. The adjacency matrix *A* $\in {\mathbb{R}}^{{\left( {r + d} \right) \times \left( {r + d} \right)}}$ of the homogeneous graph can be represented as

1 $$A = \left[ {\begin{array}{*{20}c} {SR} & {RD} \\ {RD^{T} } & {SD} \\ \end{array} } \right]$$

In this way, on the one hand, the similarity information can be used to expand the graph structure, and on the other hand, it can prevent the generation of isolated nodes during cross-validation, that is, if a node has only one edge, dividing the edge into the test set during cross-validation will cause the node to an isolated node, which makes graph convolutional network unable to learn its neighbor nodes information.

### Self-supervised learning on graphs

In recent years, due to the problems of over-fitting, poor generalization, and weak robustness of graph supervised or semi-supervised learning, self-supervised learning on graph (SSLG) has become a promising and trending learning paradigm for graph data^28,29^. In SSLG, models are learned by solving a series of handcrafted pretext tasks, in which the supervision signals are acquired from data itself automatically without the need for manual annotation. With the help of well-designed pretext tasks, SSLG enables the model to learn more informative representations from unlabeled data to achieve better performance, generalization and robustness on various downstream tasks.

Existing SSLG methods can be roughly divided into two categories: contrastive method^30–32^ and generative method^33,34^. Contrastive methods use information on commonalities and differences between data-data pairs as self-supervision signals by comparing different views. Generative methods focus on the information inside the graph data, generally based on tasks such as feature/structure reconstruction, and use the attributes and structure of the graph itself as a supervision signal. In this study, combined with the ncRNA-disease networks, we explore effective node embedding learning frameworks through above two approaches, aiming to build high-performance, generalized and robust predict models.

### Contrastive method

Contrastive method aims to learn the commonality information of different views of the same node and the difference information of different views of different nodes. Inspired by SUGRL^30^ and HCCF^31^, we implement the contrastive learning of different views through multiple strategies, and further classify them into homogeneous SSLG and heterogeneous SSLG according to the input graph structure.

**Fig. 2:**
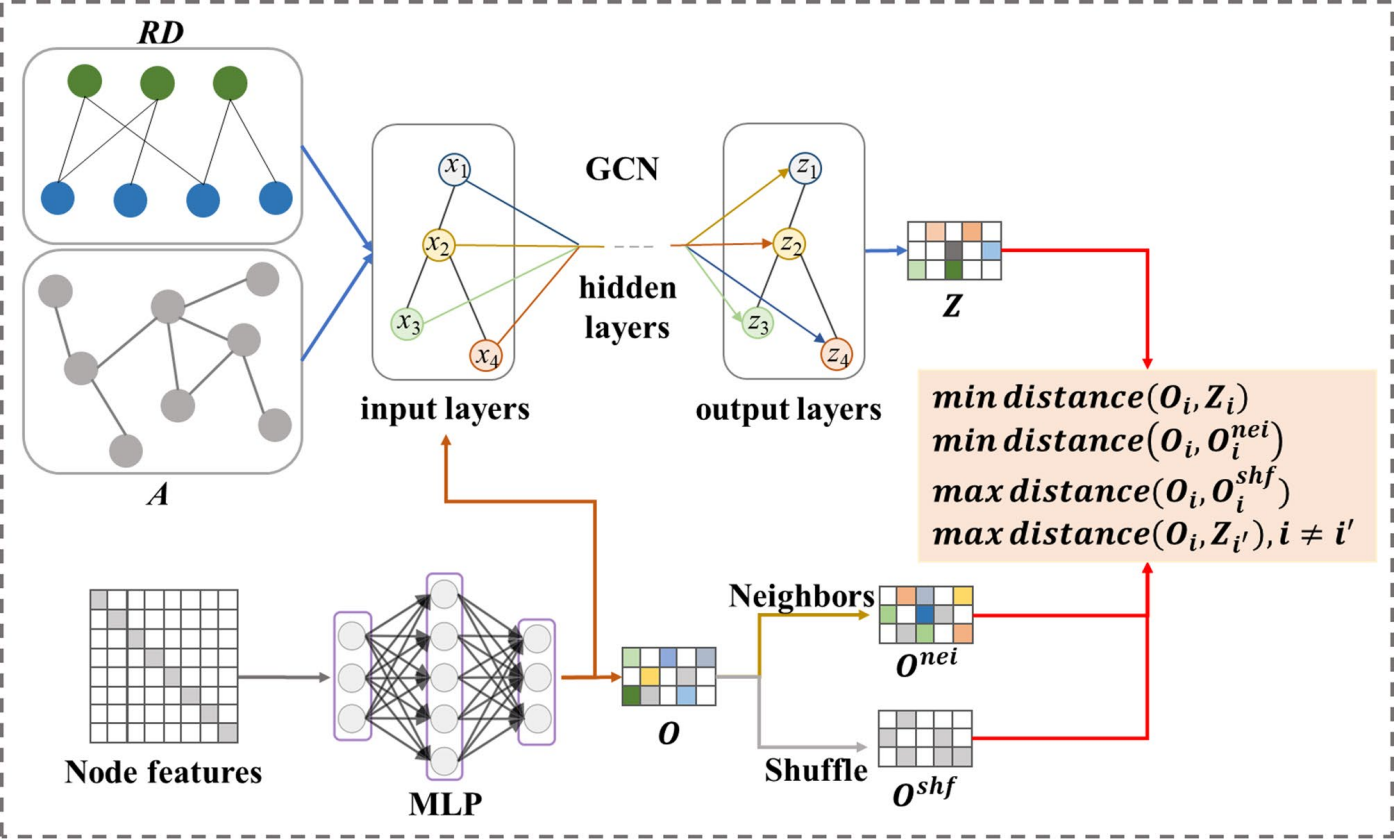
Framework of contrastive method SSLG_GM. With given graph data, GCN and MLP are applied to learn the structural features $Z$ and attribute features $O$ respectively. Randomly shuffle attribute features as negative sample features $O^{shf}$ and constructing local features $O^{nei}$ using neighborhood node features. The graph self-supervised learning model learns robust node representations by minimizing the feature distance of positive sample pairs and maximizing the feature distance of negative sample pairs.

The first strategy, we compare graph structure features and attribute features and call it SSLG_GM (see Fig. 2), which has strong scalability, computationally inexpensive, and can generate high-quality representations.

Specifically, given an ncRNA-disease homogeneous graph adjacency matrix *A* and node feature matrix *X* $\in {\mathbb{R}}^{{\left( {r + d} \right) \times \left( {r + d} \right)}}$ (composed of One-Hot Encoding), we use Multi-Layer Perceptron (MLP) and LightGCN^35^ to learn attribute feature $O$ and node structure feature $Z$, respectively,

2 $$O = \sigma \left( {XW_{o} } \right)$$

3 $$Z = \sigma \left( {\hat{A}O} \right)$$

where $W_{o} \in {\mathbb{R}}^{{\left( {r + d} \right) \times n}}$ is learnable weights matrix, $\hat{A} = D^{{ - \frac{1}{2}}} \left( {A + I} \right)D^{{ - \frac{1}{2}}}$, $D$ is the degree matrix and $I$ is the identity matrix, $\sigma \left( \cdot \right)$ denotes the ReLU activation function. $Z$ will be used for downstream tasks, and we call this sub-model SSLG_GM_homo.

For node $i$, we compute the feature representation $O_{i}^{nei}$ of its neighbors according to the adjacency matrix *A*

4 $$O_{i}^{nei} = \frac{1}{n}\mathop \sum \limits_{j = 1}^{n} O_{j} , if A_{i,j} = 1$$

where $n$ is the number of sampled neighbors. To generate negative sample views, we randomly shuffle the attribute feature matrix $O$ row-wise and denote as $O^{shf}$.

For SSLG methods, either reducing the intra-class variation or enlarging the inter-class variation has been demonstrated to be an effective solution to reduce generalization error^30^. Therefore, the multiple loss for contrastive learning between different views is formulated as

5 $${\mathcal{L}}_{1} = \frac{1}{m}\mathop \sum \limits_{i = 1}^{m} max\left\{ {0,d\left( {O,O^{nei} } \right) - d\left( {O,O_{i}^{shf} } \right) + \alpha } \right\}$$

6 $${\mathcal{L}}_{2} = \frac{1}{m}\mathop \sum \limits_{i = 1}^{m} \max \left\{ {0,d\left( {O,Z} \right) - d\left( {O,O_{i}^{shf} } \right) + \alpha } \right\}$$

7 $${\mathcal{L}}_{3} = \frac{1}{m}\mathop \sum \limits_{i = 1}^{m} \max \left\{ {0,d\left( {O,Z} \right) - d\left( {O,O_{i}^{shf} } \right) - \alpha - \beta } \right\}$$

8 $${\mathcal{L}}_{ctr} = \lambda_{1} {\mathcal{L}}_{1} + \lambda_{2} {\mathcal{L}}_{2} + \lambda_{3} {\mathcal{L}}_{3}$$

where *m* is the number of negative samples, $d\left( \cdot \right)$ is L2-norm distance measurement, $\alpha$ and $\beta$ are non-negative tuning parameters, $\lambda_{1}$, $\lambda_{2}$ and $\lambda_{3}$ are the weights of ${\mathcal{L}}_{1}$, ${\mathcal{L}}_{2}$ and ${\mathcal{L}}_{3}$, respectively. ${\mathcal{L}}_{1}$ and ${\mathcal{L}}_{2}$ are able to the enlarge inter-class variation, ${\mathcal{L}}_{3}$ can reduce intra-class variation.

Furthermore, given that node initial features are represented by one-hot encoding, adding supervision information helps to learn high-quality node embeddings. Therefore, pairwise marginal loss is introduced as the supervise signal, which is defined as

9 $${\mathcal{L}}_{sup} = \mathop \sum \limits_{i = 1}^{k} \mathop \sum \limits_{j = 1}^{s} {\mathrm{max}}\left( {0, 1 - pos_{i,j} + neg_{i,j} } \right)$$

where $k$ denotes the number of training samples, $s$ denotes the number of positive and negative samples corresponding to node *i*, *pos* and *neg* represent the prediction scores of positive and negative samples, respectively. Finally, we integrate the supervise loss ${\mathcal{L}}_{sup}$ with the contrastive loss ${\mathcal{L}}_{ctr}$ into a unified objective as

10 $${\mathcal{L}} = \lambda_{4} {\mathcal{L}}_{sup} + \lambda_{5} {\mathcal{L}}_{ctr}$$

The sub-model applied to the heterogeneous graph is called SSLG_GM_hete. SSLG_GM_hete contains two GCN modules and two MLP modules for learning feature representations for different views of ncRNA and disease, respectively. Due to some nodes in the heterogeneous graph have only one neighbor or no neighbors, we use the InfoNCE loss to train the model. The InfoNCE loss is defined as follows:

11 $${\mathcal{L}}_{rna} = \mathop \sum \limits_{i = 1}^{r} - log\frac{{{\mathrm{exp}}\left( {sim\left( {O_{i} ,Z_{i} } \right)/\tau } \right)}}{{\mathop \sum \nolimits_{{i^{\prime} = 1}}^{r} {\mathrm{exp}}\left( {sim\left( {O_{i} ,Z_{{i^{\prime}}} } \right)/\tau } \right)}}$$

12 $${\mathcal{L}}_{dis} = \mathop \sum \limits_{j = 1}^{d} - log\frac{{{\mathrm{exp}}\left( {sim\left( {O_{j} ,Z_{j} } \right)/\tau } \right)}}{{\mathop \sum \nolimits_{{j^{\prime} = 1}}^{r} {\mathrm{exp}}\left( {sim\left( {O_{j} ,Z_{{j^{\prime}}} } \right)/\tau } \right)}}$$

13 $${\mathcal{L}}_{ctr} = {\mathcal{L}}_{rna} + {\mathcal{L}}_{dis}$$

where $sim\left( \cdot \right)$ denotes the cosine similarity function and τ denotes the tunable temperature hyperparameter. The subscript $rna$ and $dis$ denote ncRNA and disease, respectively.

**Fig. 3:**
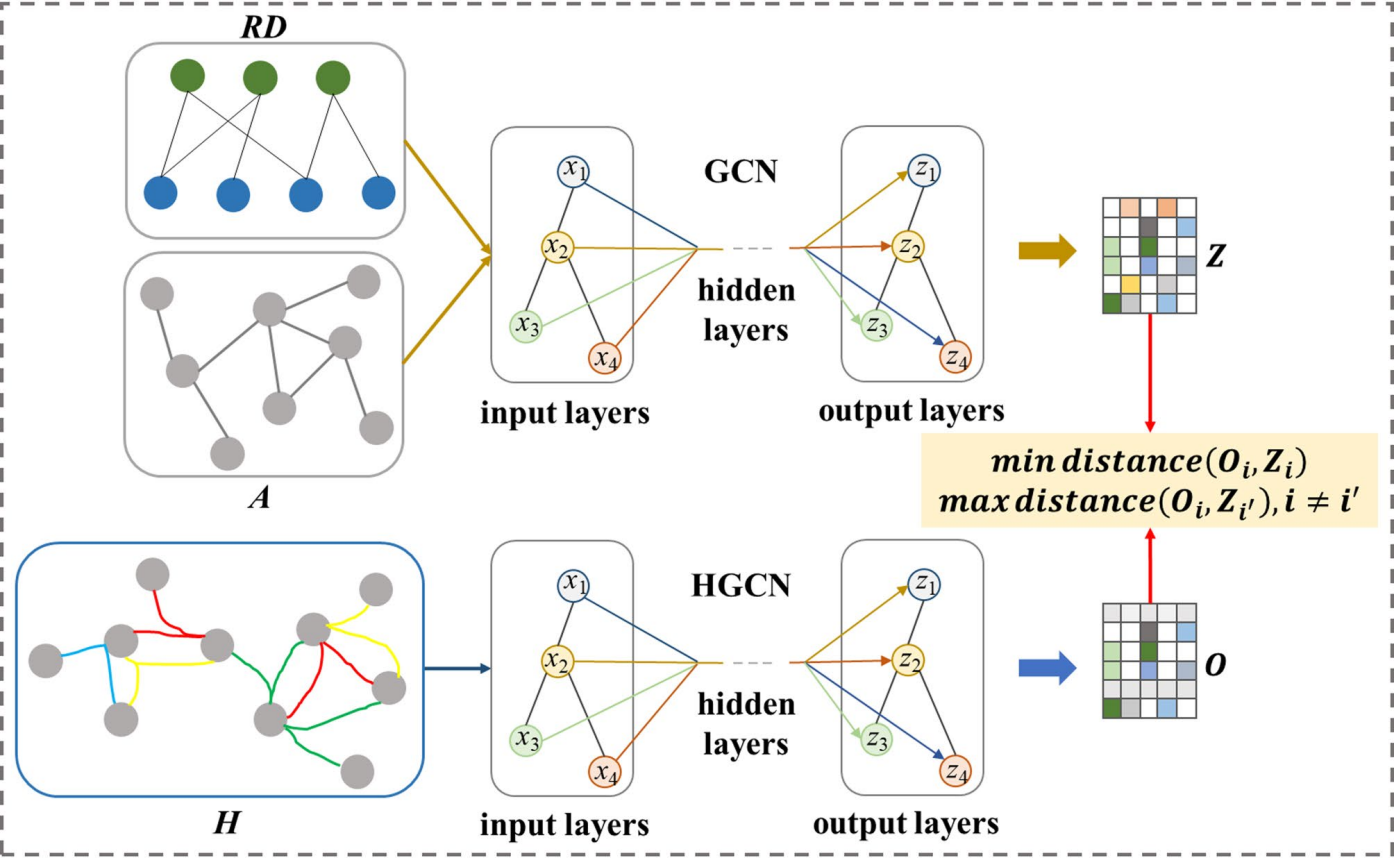
Framework of contrastive method SSLG_GH. With given graph data, GCN and HGCN are applied to learn the local structural features $Z$ and global structural features $O$ respectively.

The second strategy, we compare different graph structure features and call it SSLG_GH (see Fig. 3). SSLG_GH captures the intrinsic and implicit dependencies between ncRNA and disease through the mutual cooperative supervision between global structure and local relations. Similarly, SSLG_GH uses two types of inputs: heterogeneous graph and homogeneous graph, which we call SSLG_GH_hete and SSLG_GH_homo, respectively. Take homogeneous graph as an example, given an ncRNA-disease homogeneous graph adjacency matrix *A*, we use GCN to learn node representations and treat them as local features $Z$. At the same time, we construct a parameterized ncRNA-disease hypergraph, using a HyperGraph Convolutional Network (HGCN) to learn global features $O$ of nodes. By contrasting local features and global features, we can obtain more robust node embedding representations.

For local features, we also use LightGCN to learn node embeddings

14 $$Z = \sigma \left( {\hat{A}E} \right)$$

where $E \in {\mathbb{R}}^{{\left( {r + d} \right) \times e}}$ denotes the learnable parameterized feature matrix, $e$ represents node embedding dimension.

To adaptively learn hypergraph-based dependent structures across nodes, we use parameterized hypergraph structure learning to obtain global features. Parameterized hypergraph structure is defined as below:

15 $$H = E \cdot W_{H}$$

where $W_{H} \in {\mathbb{R}}^{e \times h}$ represents the learnable embedding matrices for hyperedges, $h$ denotes the number of hyperedges. In order to further supercharge hypergraph neural architecture with a high-level of hyperedge-wise feature interaction, different layers of hyperedges are stacking as follow:

16 $$F^{\left( 0 \right)} = H^{T} \cdot E$$

17 $$F^{\left( l \right)} = \sigma \left( {W^{{\left( {l - 1} \right)}} F^{{\left( {l - 1} \right)}} } \right) + F^{{\left( {l - 1} \right)}}$$

where $W^{{\left( {l - 1} \right)}} \in {\mathbb{R}}^{h \times h}$ is a trainable parametric matrix, $l$ denotes the number of hypergraph embedding layers. After the hierarchical hypergraph mapping, we refine the global features:

18 $$O = \sigma \left( {HF^{\left( l \right)} } \right)$$

Same as SSLG_GM, SSLG_GH_homo is trained with triplet loss and SSLG_GH_hete is trained with InfoNCE loss. Finally, we integrate the task loss and the auxiliary constraints to form the overall loss, which is optimized by

19 $${\mathcal{L}} = \lambda_{1} {\mathcal{L}}_{sup} + \lambda_{2} {\mathcal{L}}_{ctr} + \lambda_{3} {\Theta }_{F}^{2}$$

where ${\Theta }$ denotes the weight-decay regularization term.

### Generative method

**Fig. 4:**
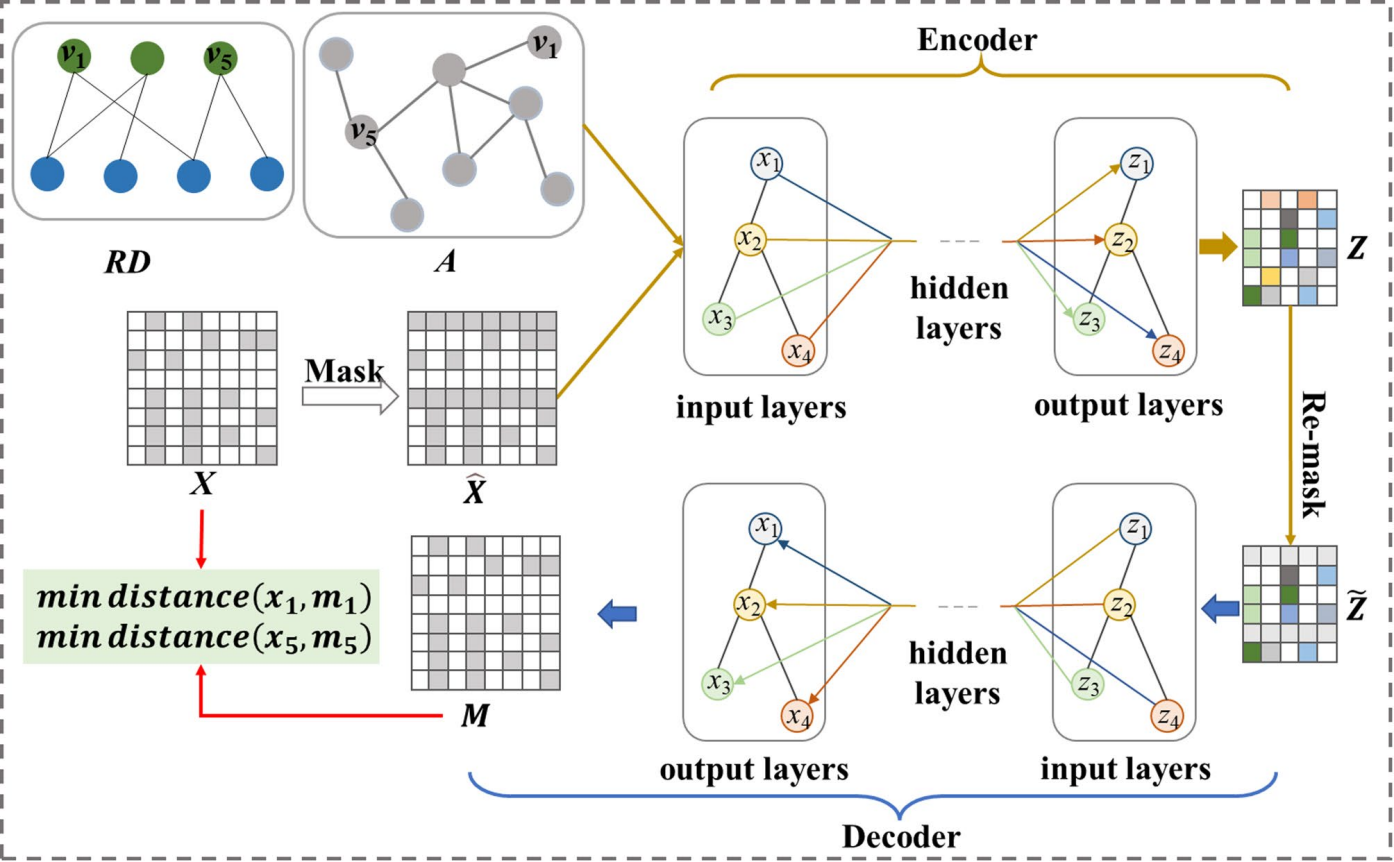
Framework of generative method SSLG_MA. Given the graph data and node features, the node features are masked randomly, and the masked features are reconstructed by using GCN encoder and decoder, and effective node representations are learned by comparing the differences between the original features and the reconstructed features.

Contrastive learning has been the dominant approach in SSLG, while the progress of generative SSLG has thus far not reached its true potential. Generative method aims to learn robust node embeddings by reconstructing graph structure or node attributes. Inspired by GraphMAE^33^, we introduce a self-supervised learning method SSLG_MA for node attribute reconstruction, whose node embedding module consists of a GCN encoder and a GCN decoder (see Fig. 4). SSLG_MA uses a masking strategy to achieve feature reconstruction, which is beneficial to obtain robust node embeddings. We also divide SSLG_MA into SSLG_MA_homo and SSLG_MA_hete. To the best of our knowledge, this is the first attempt that semantic features have been reconstructed in a graph self-supervised manner in RNA-disease association prediction. Here, semantic features refer to the intrinsic biological attributes of nodes (represented by the similarity feature matrix derived from sequence or functional data), as opposed to the structural features represented by the graph adjacency matrix. By reconstructing these attributes, the model is encouraged to capture the underlying biological context of ncRNAs and diseases beyond their topological connections.

Given an ncRNA-disease homogeneous graph adjacency matrix *A* and node feature matrix *X* (consist of node similarity), a set of nodes $\tilde{v}$ (such as $v_{1} ,v_{5}$) will be randomly selected with their initial features replaced by learnable vectors $x^{\prime}$(such as $x_{1}{\prime} ,x_{5}{\prime}$). Thus, the new feature representation of the node $v_{i}$ is as follows,

20 $$\hat{x}_{{v_{i} }} = \left\{ {\begin{array}{*{20}c} {x_{{v_{i} }}{\prime} v_{i} \in \tilde{v}} \\ {x_{{v_{i} }} others} \\ \end{array} } \right.$$

The encoder maps the features $\hat{X}$ to the latent space and denoted by $Z$,

21 $$Z = En\left( {A,\hat{X}} \right)$$

where $En\left( \cdot \right)$ denotes the GCN encoder.

To further encourage the encoder to learn compressed representations, the representations of node $v_{i} \in \tilde{v}$ in $Z$ are again replaced by another learnable vectors $z_{i}^{\prime }$, the re-masked node representations is denoted as:

22 $$\tilde{z}_{{v_{i} }} = \left\{ {\begin{array}{*{20}c} {z_{{v_{i} }}{\prime} v_{i} \in \tilde{v}} \\ {z_{{v_{i} }} others} \\ \end{array} } \right.$$

Then, the decoder is to reconstruct the input as

23 $$M = De\left( {A,\tilde{Z}} \right)$$

where $De\left( \cdot \right)$ denotes the GCN decoder.

To obtain node representations that are beneficial for association prediction, InfoNCE loss is used to train SSLG_MA model. On the one hand, it reduces the distance between the reconstructed feature and the original feature, and on the other hand, it increases the difference be-tween the features of different nodes. The loss function is defined as:

24 $$L = \mathop \sum \limits_{i = 1}^{{\left| {\tilde{v}} \right|}} - \log \frac{{\exp \left( {sim\left( {Z_{i} ,M_{i} } \right)/\tau } \right)}}{{\mathop \sum \nolimits_{i\prime = 1}^{{\left| {\tilde{v}} \right|}} \exp \left( {sim\left( {Z_{i} ,M_{i\prime } } \right)/\tau } \right)}}$$

where $\left| {\tilde{v}} \right|$ indicates the number of nodes to be masked. For downstream task, the encoder is applied to the input graph without any masking. The generated node embeddings $Z$ are used for association prediction.

### Link prediction

With the SSLG algorithm, we obtained robust node embeddings. Given a known ncRNA-disease pair, its features are denoted as $Fea = [Z_{rna} ||Z_{dis} ]$. The notation $||$ means concatenation. Based on $Fea$, we train Extra-Trees (ET) as predictors. For unknown ncRNA-disease pairs, we inferred their association scores by ET. Higher scores for ncRNA-disease pairs indicate that ncRNAs are more likely to be disease-related.

Overall, our SSLGRDA framework mainly explores graph self-supervised learning algorithms that can learn inherent, transferable, and robust knowledge in graph data from unlabeled data. This architecture has a wide range of application scenarios: RDA graphs can be either heterogeneous graphs or homogeneous graphs, and node attribute features can be composed of similarities between nodes or one-hot encoding. More importantly, the architecture has good prediction accuracy.

## Results

### Evaluation protocols and metrics

We adopt five-fold cross-validation (5-CV) to evaluate the performance of prediction models. The known associations are equally divided into 5 parts, 4 parts are used to build the graph, and the remaining one part is used for testing. Specifically, first, the graph of training data is utilized to learn node embeddings and the corresponding links are used to train a binary logistic classifier. The training set consists of 4/5 of the known associations and the same number of unknown associations. Then, test relations with a set of random negative (non-connected) links are used to evaluate the trained classifier. The test set consists of 1/5 of the known associations and 1/5 of the unknown association.

All models were implemented using the PyTorch framework and trained on an NVIDIA GeForce RTX 3090 GPU. The models were trained for 100 epochs using the Adam optimizer. Through grid search, we determined the optimal hyperparameters for each sub-model.

For SSLG_GM, the learning rate is set to 0.005 with a weight decay of 1e-4. The MLP hidden layer size is 128. The contrastive loss weights are set to ${\uplambda }_{{1}} { = 5}$, ${\uplambda }_{{2}} { = 5}$ and ${\uplambda }_{{3}} { = 1}$, with tuning parameters $\alpha = 0.8$ and $\beta = 0.4$. The Task weight ${\uplambda }_{{4}}$ of supervise loss is 1e-5, the task weight ${\uplambda }_{{5}}$ of contrastive loss is 1.

For SSLG_GH, the learning rate is 1e-3. The node embedding dimension is 128. The hypergraph structure is defined by 50 hyperedges (*h* = 50) and 2 embedding layers (*l* = 2).

For SSLG_MA, the learning rate is 0.005. The GCN encoder consists of 2 layers with 128 hidden units. The masking rate is set to 0.4, and the temperature parameter ${\uptau }$ for InfoNCE is 0.9.

Treating ncRNA-disease association prediction as a binary classification task, we adopt three classification evaluation metrics to evaluate the performances of our model, i.e., the area under receiver-operating characteristic curve (AUC), the area under the precision-recall curve (AUPR) and F1 score, the higher the value, the better the model performance.

Drawing on the evaluation metrics of link prediction in knowledge graph, we introduced ranking metrics, namely Mean Rank (MR), Mean Reciprocal Ranking (MRR), and Hits@N. MR means that each positive and all negative samples in the test set are sorted in descending order according to the prediction score, and the average ranking of all positive samples is calculated according to the following formula

25 $${\mathrm{MR}} = \frac{1}{\left| S \right|}\left( {rank_{1} + rank_{2} + \ldots + rank_{\left| S \right|} } \right)$$

where $rank_{i}$ is the rank of the *i*-th positive sample, $\left| S \right|$ is the number of positive samples. MRR represents the average value of the reciprocal of the ranking of positive samples, and can be calculated by

26 $${\mathrm{MRR}} = \frac{1}{\left| S \right|}\left( {\frac{1}{{rank_{1} }} + \frac{1}{{rank_{2} }} + \ldots + \frac{1}{{rank_{\left| S \right|} }}} \right)$$

Hits@N represents the ratio of the number of top *N* positive samples to the number of all positive samples, the formula is expressed as

27 $${\mathrm{Hits}}@{\mathrm{N}} = \frac{1}{\left| S \right|}\mathop \sum \limits_{i = 1}^{\left| S \right|} \left| {\left( {rank_{i} \le N} \right)} \right.$$

where $\left| {\left( \cdot \right)} \right.$ is the indicator function (if the condition is true, the function value is 1, otherwise it is 0). It should be noted that the smaller the MR is, the better the model performance is, and the larger the MRR and Hits@N are, the better the model performance is.

Moreover, we also locally ranked each RNA-related diseases and each disease-related ncRNAs in the test set to simulate specific retrieval tasks. Specifically, for MR_L_R (and MRR_L_R), we grouped the test samples by disease; for each disease, we ranked the true associated ncRNA against the negative ncRNAs paired with that specific disease. Similarly, for MR_L_D (and MRR_L_D), we grouped samples by ncRNA and ranked the true associated disease against the negative diseases paired with that specific ncRNA. These metrics were calculated as the Mean Rank and Mean Reciprocal Ranking within these local groups, where L represents local, R and D represent ncRNA and disease, respectively.

### Baseline methods

Based on SSL contrastive methods, we proposed SSLG_GH and SSLG_GM sub-models, and based on SSL generative method, we proposed SSLG_MA sub-model. In addition, based on the input graph structure, the above models are further divided into homogeneous graph models: SSLG_GH_homo, SSLG_GM_ homo, SSLG_MA_ homo and heterogeneous graph models: SSLG_GH_hete, SSLG_GM_hete, SSLG_MA_hete.

To comprehensively evaluate the performance of SSLGRDA, we selected representative baseline methods based on their relevance to our methodological contributions, code availability, and wide recognition in the field. These methods cover three distinct categories to benchmark different aspects of our framework. Two SSL algorithms: AFGRL^32^ and GAE^34^, four ncRNA-Disease Association Prediction algorithms (RDAP): LR-GNN^17^, MINIMDA^19^, GMNN2CD^18^ and MLGCN^36^, three heterogenous Graph Neural Networks algorithms (HeteGNN): RGCN^37^, GATNE^38^ and HGB^39^. In particular, AFGRL is a contrastive method (SSLG_Con), which generates a second view by discovering nodes that share local structural information and global semantics with the original graph, and then performs contrastive learning to obtain node embedding. GAE is a generative method (SSLG_Gen) that reconstructs the structure of the graph through the encoder-decoder to obtain node embeddings.

### Results for circRNA-disease datasets

Table 2, Supplementary Table 1 and Supplementary Table 2 summarize classification accuracy and ranking results of all methods on three circRNA-disease datasets, where the best results are highlighted in bold. In general, SSLG_GM_homo performs best on CDA1 and CDA2, SSLG_GH_homo performs best on CDA3, and second-best on CDA1 and CDA2. For other models, there may be one or more best metrics, such as GATNE has the best MRR on CDA1 and the best F1 on CDA2.

**Table 2:** Classification accuracy and ranking results of all methods on CDA1.

Dataset	Category	Model	AUC	AUPR	F1	Hits@10	Hits@50	Hits@100
CDA1	Contrastive	SSLG_GH_hete	0.72292	0.01710	0.01947	0.00645	0.02258	0.04839
	Contrastive	SSLG_GH_homo	*0.94676*	*0.72611*	*0.42944*	*0.53846*	*0.72615*	*0.80462*
	Contrastive	SSLG_GM_hete	0.69728	0.01715	0.00044	0.00516	0.02000	0.04032
	Contrastive	SSLG_GM_homo	**0.95895**	**0.83201**	**0.57952**	**0.68969**	**0.84600**	**0.85708**
	Generative	SSLG_MA_hete	0.71561	0.01846	0.02698	0.01613	0.02581	0.04516
	Generative	SSLG_MA_homo	0.93966	0.30896	0.17583	0.09846	0.31077	0.41231
	SSLG_Con	AFGRL	0.86479	0.37018	0.17264	0.17692	0.30000	0.41538
	SSLG_Gen	GAE	0.93717	0.72373	0.34548	0.52769	0.68462	0.76923
	RDAP	LR-GCN_hete	0.79853	0.05263	0.06569	0.01339	0.07839	0.12365
	RDAP	LR-GCN_homo	0.70420	0.03922	0.04983	0.00615	0.03846	0.06308
	RDAP	GMNN2CD	0.91405	0.31032	0.03586	0.22154	0.23385	0.28308
	RDAP	MINIMDA	0.66761	0.02512	0.05008	0.00077	0.00308	0.01462
	RDAP	MLGCN	0.88863	0.16228	0.12119	0.05385	0.13231	0.21077
	HeteGNN	GATNE	0.89522	0.71146	0.36170	0.57692	0.66154	0.74615
	HeteGNN	HGB	0.80291	0.16013	0.05839	0.06154	0.17692	0.23846
	HeteGNN	RGCN	0.78827	0.08469	0.14498	0.02615	0.06615	0.11231
Dataset	Category	Model	MR↓	MRR	MR_L_R↓	MR_L_D↓	MRR_L_R	MRR_L_D
CDA1	Contrastive	SSLG_GH_hete	2845.77	0.00284	9.25635	40.02818	0.25703	0.16390
	Contrastive	SSLG_GH_homo	*505.54*	0.21271	**1.75365**	*9.49539*	*0.89103*	*0.66816*
	Contrastive	SSLG_GM_hete	3135.59	0.00410	9.00988	40.03564	0.25463	0.14122
	Contrastive	SSLG_GM_homo	**447.12**	*0.38614*	*2.04862*	**7.20235**	**0.89365**	**0.72038**
	Generative	SSLG_MA_hete	2869.97	0.00505	8.44495	40.22265	0.26071	0.16896
	Generative	SSLG_MA_homo	614.74	0.04422	2.19443	10.48246	0.78697	0.43954
	SSLG_Con	AFGRL	1361.11	0.09458	2.94048	21.23905	0.75261	0.56210
	SSLG_Gen	GAE	633.29	0.38549	2.26168	11.09363	0.86842	0.61310
	RDAP	LR-GCN_hete	1013.19	0.00838	3.54970	19.80654	0.49511	0.19655
	RDAP	LR-GCN_homo	2978.08	0.00480	5.40351	48.79999	0.37175	0.07248
	RDAP	GMNN2CD	874.76	0.15721	4.59866	14.84050	0.64709	0.29004
	RDAP	MINIMDA	3345.46	0.00121	5.27989	73.32454	0.43707	0.02757
	RDAP	MLGCN	1125.89	0.03146	3.09892	18.01503	0.65333	0.27742
	HeteGNN	GATNE	1066.20	**0.50440**	2.10484	19.68520	0.83991	0.58592
	HeteGNN	HGB	2004.59	0.03737	3.63095	30.50231	0.54251	0.10612
	HeteGNN	RGCN	2153.42	0.01846	4.25318	29.65993	0.51558	0.14081

↓ means the smaller the better. Best results in the experiment are highlighted in bold, and the second-best result is italic.

### Results for lncRNA-disease datasets

The averaged results of all methods on three lncRNA-disease datasets are reported in Table 3 Supplementary Table 3 and Supplementary Table 4. In summary, SSLG_GH_homo significantly outperforms the other models on LDA1 and LDA3, SSLG_GM_homo performs second-best on LDA1 and LDA3. On LDA2, SSLG_MA_hete has the best classification accuracy and Hits@N score, followed by SSLG_GH_hete.

**Table 3:** Classification accuracy and ranking results of all methods on LDA1.

Dataset	Category	Model	AUC	AUPR	F1	Hits@10	Hits@50	Hits@100
LDA1	Contrastive	SSLG_GH_hete	0.96566	0.61504	0.39035	0.17321	0.36842	0.49234
	Contrastive	SSLG_GH_homo	**0.98653**	**0.73345**	**0.41354**	**0.24242**	**0.50080**	**0.64833**
	Contrastive	SSLG_GM_hete	0.95529	0.49556	0.29238	0.13014	0.26746	0.36172
	Contrastive	SSLG_GM_homo	*0.98612*	*0.71603*	*0.40266*	0.20268	*0.45823*	*0.59067*
	Generative	SSLG_MA_hete	0.93885	0.31573	0.22026	0.02196	0.10866	0.20349
	Generative	SSLG_MA_homo	0.95592	0.43262	0.26116	0.19234	0.22967	0.32679
	SSLG_Con	AFGRL	0.97617	0.62338	0.34223	0.18660	0.36364	0.48325
	SSLG_Gen	GAE	0.97684	0.58837	0.35696	0.16220	0.31675	0.43923
	RDAP	LR-GCN_hete	0.92638	0.34157	0.12905	0.08133	0.16478	0.23286
	RDAP	LR-GCN_homo	0.87691	0.29530	0.18583	0.05646	0.13732	0.21435
	RDAP	GMNN2CD	0.71003	0.28205	0.05923	*0.21483*	0.26172	0.27033
	RDAP	MINIMDA	0.91548	0.34152	0.22909	0.08852	0.19019	0.24282
	RDAP	MLGCN	0.98524	0.71019	0.40172	0.20063	0.45250	0.58150
	HeteGNN	GATNE	0.84256	0.11378	0.16690	0.00478	0.03110	0.05502
	HeteGNN	HGB	0.87848	0.20765	0.16610	0.05263	0.09330	0.12440
	HeteGNN	RGCN	0.87749	0.19066	0.24181	0.00861	0.04880	0.08612
Dataset	Category	Model	MR↓	MRR	MR_L_R↓	MR_L_D↓	MRR_L_R	MRR_L_D
LDA1	Contrastive	SSLG_GH_hete	595.30	0.11068	6.26541	4.03184	0.56028	0.63097
	Contrastive	SSLG_GH_homo	**201.23**	*0.11949*	**2.85729**	**1.99521**	*0.72039*	**0.77153**
	Contrastive	SSLG_GM_hete	810.86	0.08588	7.45672	4.11368	0.51569	0.56362
	Contrastive	SSLG_GM_homo	*260.47*	0.09329	*3.06669*	*2.15334*	**0.72189**	*0.75809*
	Generative	SSLG_MA_hete	1109.07	0.01313	9.88174	5.33558	0.44566	0.49817
	Generative	SSLG_MA_homo	792.75	**0.19660**	8.88606	4.40137	0.47160	0.52967
	SSLG_Con	AFGRL	424.78	0.08636	4.14473	2.72629	0.67131	0.68541
	SSLG_Gen	GAE	412.88	0.09001	4.57067	2.51083	0.62589	0.68842
	RDAP	LR-GCN_hete	1309.43	0.04632	11.33487	6.68853	0.39636	0.44263
	RDAP	LR-GCN_homo	2189.63	0.03386	15.14383	9.33904	0.35591	0.41329
	RDAP	GMNN2CD	5252.06	0.10525	8.44548	18.31646	0.54207	0.15131
	RDAP	MINIMDA	1503.19	0.04376	14.27812	6.20406	0.36108	0.48175
	RDAP	MLGCN	268.50	0.11748	3.72657	2.22322	0.68498	0.72732
	HeteGNN	GATNE	2852.10	0.00292	11.85568	9.28514	0.34212	0.32491
	HeteGNN	HGB	2201.59	0.02204	8.95057	7.74927	0.46247	0.34281
	HeteGNN	RGCN	2219.45	0.00860	16.93909	8.26660	0.28875	0.35444

↓ means the smaller the better. Best results in the experiment are highlighted in bold, and the second-best result is italic.

### Results for miRNA-disease datasets

The performances of all models on three miRNA-disease datasets are reported in Table 4, Supplementary Table 5 and Supplementary Table 6, respectively. In a word, SSLG_GM_homo has the best Hits@50, 100 score and local ranking on MDA1, and has the best classification accuracy on MDA2 and MDA3. SSLG_GH_homo has the second-best classification accuracy on MDA3.

**Table 4:** Classification accuracy and ranking results of all methods on MDA1.

Dataset	Category	Model	AUC	AUPR	F1	Hits@10	Hits@50	Hits@100
MDA1	Contrastive	SSLG_GH_hete	**0.93883**	0.46842	0.30549	*0.04309*	*0.13444*	0.20773
	Contrastive	SSLG_GH_homo	0.92693	0.44592	0.28615	0.04880	0.14487	0.22376
	Contrastive	SSLG_GM_hete	*0.93399*	0.46367	**0.30804**	0.05009	0.15543	0.21584
	Contrastive	SSLG_GM_homo	0.93122	**0.47029**	0.29192	0.05514	**0.16041**	**0.23396**
	Generative	SSLG_MA_hete	0.93108	0.44375	0.30071	0.04770	0.13020	0.19797
	Generative	SSLG_MA_homo	0.91678	0.39178	0.24247	**0.10645**	0.11952	0.18195
	SSLG_Con	AFGRL	0.92234	0.46117	0.28310	0.05709	0.15746	*0.22468*
	SSLG_Gen	GAE	0.91513	0.39618	0.26481	0.04862	0.10331	0.16943
	RDAP	LR-GCN_hete	0.92004	0.38672	0.20767	0.03597	0.10434	0.16088
	RDAP	LR-GCN_homo	0.88110	0.32691	0.21098	0.02192	0.08527	0.14291
	RDAP	GMNN2CD	0.81397	0.28331	0.03666	0.07053	*0.15985*	0.20534
	RDAP	MINIMDA	0.89660	0.37710	0.29534	0.03573	0.10534	0.15580
	RDAP	MLGCN	0.92352	0.43750	0.28659	0.07845	0.14236	0.20589
	HeteGNN	GATNE	0.72438	0.06313	0.05808	0.00092	0.00276	0.00737
	HeteGNN	HGB	0.87214	0.18775	0.18546	0.00460	0.02026	0.04972
	HeteGNN	RGCN	0.80566	0.14603	0.05733	0.00258	0.01510	0.03057
Dataset	Category	Model	MR↓	MRR	MR_L_R↓	MR_L_D↓	MRR_L_R	MRR_L_D
MDA1	Contrastive	SSLG_GH_hete	**2199.96**	0.02537	**8.36475**	15.94755	0.40922	*0.31673*
	Contrastive	SSLG_GH_homo	2749.87	0.03146	9.94598	16.17752	*0.42686*	0.30483
	Contrastive	SSLG_GM_hete	*2437.27*	0.02616	9.07621	*15.80073*	0.39883	0.31493
	Contrastive	SSLG_GM_homo	2478.45	0.02826	8.97314	**15.29372**	**0.42860**	**0.33502**
	Generative	SSLG_MA_hete	2545.19	0.02691	9.28534	16.60946	0.40559	0.31130
	Generative	SSLG_MA_homo	3066.78	**0.10892**	10.80616	17.62210	0.38889	0.28082
	SSLG_Con	AFGRL	2794.09	0.02541	9.22046	16.88536	0.42161	0.30959
	SSLG_Gen	GAE	3053.89	0.04886	10.29187	16.80459	0.38213	0.29028
	RDAP	LR-GCN_hete	2876.55	0.01668	9.68723	17.98385	0.36198	0.21618
	RDAP	LR-GCN_homo	4279.57	0.01331	12.05785	24.41551	0.36925	0.18333
	RDAP	GMNN2CD	6852.57	0.03329	12.39874	38.40144	0.39120	0.09914
	RDAP	MINIMDA	3719.30	0.01604	10.00389	16.86231	0.38459	0.23586
	RDAP	MLGCN	2817.91	*0.08268*	9.76034	16.38038	0.39902	0.29795
	HeteGNN	GATNE	10,152.23	0.00071	30.48260	28.81425	0.07739	0.11567
	HeteGNN	HGB	4710.20	0.00277	10.53980	24.00920	0.36527	0.18400
	HeteGNN	RGCN	7158.69	0.00339	18.82984	28.92183	0.22460	0.13941

↓ means the smaller the better. Best results in the experiment are highlighted in bold, and the second-best result is italic.

Furthermore, to validate the statistical significance of these results, we performed paired t-tests on the fivefold cross-validation outputs. Supplementary Table 7 indicate that the improvements of SSLGRDA over the second-best methods are statistically significant with p < 0.05 in terms of AUC and AUPR.

All in all, based on the experimental results and data characteristics, we have the following observations and analyses: (a)The contrastive strategy generally outperforms the generative strategy. This is likely because contrastive learning optimizes for discriminative representations by maximizing mutual information between views, which directly benefits the downstream binary classification task. In contrast, generative methods focus on feature reconstruction, which may force the model to encode noise present in the raw similarity data. (b)The homogeneous graph variants (SSLG_homo) often perform slightly better than heterogeneous ones. This can be attributed to our graph construction strategy (Section "NcRNA-disease homogeneous graph"), where similarity matrices are integrated as edges in the homogeneous graph. This densifies the sparse ncRNA-disease network, allowing GCNs to aggregate information more effectively than in the heterogeneous setting where relation types are strictly separated. (c)Among the sub-models, SSLG_GM (Graph-MLP contrast) demonstrates high robustness. By contrasting the topological view (GCN) with the attribute view (MLP), SSLG_GM effectively captures both the local structural context and the intrinsic semantic similarity of the nodes. Finally, our proposed approach significantly outperforms the existing RDAP and HeteGNN algorithms, demonstrating that self-supervised pre-training provides a more generalizable initialization for link prediction than purely supervised end-to-end training.

### Performance on other datasets

To illustrate the potential generalization of our model, we applied SSLG_GM_homo and SSLG_GH_homo to other real-world networks: microbe-disease associations (MeDiA)^40^ and microbe-drug associations (MeDrA)^41^. MeDiA contains 2 subnets of HMDAD and Disbiome, and MeDrA contains 3 subnets of MDAD, aBiofilm and DrugVirus. The fivefold CV was implemented according to GATMDA and GCNMDA. Table 5 records the results on MeDiA datasets. Table 6 reports the results on MeDrA datasets. The results show that both SSLG_GM_homo and SSLG_GH_homo outperform all baseline models.

**Table 5:** The summary of model performance on MeDiA datasets.

Methods	HMDAD		Disbiome	
	AUC	AUPR	AUC	AUPR
^BiRWHMDA	0.8890 $\pm$ 0.0194	0.8969 $\pm$ 0.0146	0.8344 $\pm$ 0.0089	0.8104 $\pm$ 0.0103
^NGRHMDA	0.8921 $\pm$ 0.0327	0.9062 $\pm$ 0.0268	0.8313 $\pm$ 0.0052	0.8202 $\pm$ 0.0043
^BRWMDA	0.8916 $\pm$ 0.0029	0.9064 $\pm$ 0.0152	0.8266 $\pm$ 0.0031	0.8031 $\pm$ 0.0041
^GRNMFMDA	0.8806 $\pm$ 0.0156	0.8914 $\pm$ 0.0162	0.8609 $\pm$ 0.0047	0.8669 $\pm$ 0.0060
^GATMDA	0.9554 $\pm$ 0.0184	0.9334 $\pm$ 0.0417	0.9307 $\pm$ 0.0079	0.9211 $\pm$ 0.0088
SSLG_GM_homo	*0.9676 *$\pm$* 0.0115*	*0.9737 *$\pm$* 0.0088*	*0.9330 *$\pm$* 0.0046*	*0.9375 *$\pm$* 0.0050*
SSLG_GH_homo	**0.9692** $\pm$ **0.0056**	**0.9739** $\pm$ **0.0046**	**0.9508** $\pm$ **0.0034**	**0.9488** $\pm$ **0.0078**

′^′ indicates that the results for these models are from GATMDA. Best results in the experiment are highlighted in bold, and the second-best result is italic.

**Table 6:** The summary of model performance on MeDrA datasets.

Methods	MDAD		aBiofilm		DrugVirus	
	AUC	AUPR	AUC	AUPR	AUC	AUPR
~ WMGHMDA	0.866 $\pm$ 0.012	0.838 $\pm$ 0.008	0.845 $\pm$ 0.006	0.890 $\pm$ 0.006	0.723 $\pm$ 0.021	0.769 $\pm$ 0.022
~ GCMDR	0.849 $\pm$ 0.006	0.851 $\pm$ 0.004	0.877 $\pm$ 0.008	0.885 $\pm$ 0.006	0.824 $\pm$ 0.017	0.821 $\pm$ 0.014
~ BLM-NII	0.923 $\pm$ 0.017	0.926 $\pm$ 0.015	0.926 $\pm$ 0.084	0.934 $\pm$ 0.063	0.891 $\pm$ 0.019	0.892 $\pm$ 0.022
~ WNN-GIP	0.872 $\pm$ 0.016	0.892 $\pm$ 0.014	0.902 $\pm$ 0.019	0.941 $\pm$ 0.013	0.800 $\pm$ 0.019	0.844 $\pm$ 0.018
~ GCNMDA	0.942 $\pm$ 0.011	0.938 $\pm$ 0.011	0.952 $\pm$ 0.003	0.949 $\pm$ 0.003	0.899 $\pm$ 0.031	0.904 $\pm$ 0.037
SSLG_GM_homo	*0.976 *$\pm$* 0.005*	*0.981 *$\pm$* 0.004*	*0.986 *$\pm$* 0.003*	*0.988 *$\pm$* 0.002*	*0.910 *$\pm$* 0.018*	*0.911 *$\pm$* 0.020*
SSLG_GH_homo	**0.983** $\pm$ **0.004**	**0.987** $\pm$ **0.005**	**0.990** $\pm$ **0.003**	**0.991** $\pm$ **0.002**	**0.917** $\pm$ **0.019**	**0.916** $\pm$ **0.021**

′ ~ ′ indicates that the results for these models are from GCNMDA. Best results in the experiment are highlighted in bold, and the second-best result is italic.

### Case studies

To further evaluate the performance of SSLGRDA, we demonstrate its ability to predict new ncRNA-disease associations. We conduct case studies on MDA1, CDA1 and LDA1 dataset. All known ncRNA-disease associations are used to train the SSLGRDA model, and all unknown ncRNA-disease pairs are used as the candidate ncRNA-disease associations for prediction. After prediction by SSLGRDA model, given a specific disease, we rank those candidate ncRNAs based on their prediction scores. For MDA and LDA, we select Breast Cancer and Colon Cancer to predict new disease-associated ncRNA. The top 15 prediction results have been validated by public databases: dbDEMC^42^, miR2disease^43^, Lnc2Cancer^44^, LncRNADisease^45^, and published literature (see Tables 7 and 8). For CDA, we selected 10 ncRNA-disease pairs out of the top 15, and validated the predictions of each pair by searching the published literature (see Table 9).

**Table 7:** The top 15 breast cancer-related candidate ncRNAs.

Rank	lncRNA	Evidence	miRNA	Evidence
1	CDKN2B-AS1	LncRNADisease	hsa-mir-106a	dbDEMC
2	MEG3	Lnc2Cancer&LncRNADisease	hsa-mir-130a	dbDEMC
3	MIR17HG	Unknown	hsa-mir-150	dbDEMC
4	H19	Lnc2Cancer&LncRNADisease	hsa-mir-142	PMID: 33,785,332
5	MALAT1	Lnc2Cancer&LncRNADisease	hsa-mir-99a	dbDEMC
6	KCNQ1OT1	Lnc2Cancer&LncRNADisease	hsa-mir-98	dbDEMC&miR2disease
7	HOTAIR	Lnc2Cancer&LncRNADisease	hsa-mir-138	dbDEMC
8	TP53COR1	LncRNADisease	hsa-mir-99b	dbDEMC
9	PVT1	Lnc2Cancer&LncRNADisease	hsa-mir-192	dbDEMC
10	GAS5	Lnc2Cancer&LncRNADisease	hsa-mir-196b	dbDEMC
11	MIAT	Lnc2Cancer&LncRNADisease	hsa-mir-15b	dbDEMC
12	ESRG	Unknown	hsa-mir-449a	PMID: 30,488,443
13	NEAT1	Lnc2Cancer&LncRNADisease	hsa-mir-212	dbDEMC
14	XIST	Lnc2Cancer&LncRNADisease	hsa-mir-185	dbDEMC
15	UCA1	Lnc2Cancer&LncRNADisease	hsa-mir-130b	dbDEMC

**Table 8:** The top 15 colon cancer-related candidate ncRNAs.

Rank	lncRNA	Evidence	miRNA	Evidence
1	H19	Lnc2Cancer&LncRNADisease	hsa-mir-21	dbDEMC&miR2disease
2	MEG3	Lnc2Cancer&LncRNADisease	hsa-mir-20a	dbDEMC&miR2disease
3	TP53COR1	Unknown	hsa-mir-155	dbDEMC&miR2disease
4	MALAT1	Lnc2Cancer&LncRNADisease	hsa-mir-18a	dbDEMC&miR2disease
5	MIR17HG	PMID: 35,116,852	hsa-mir-19b	dbDEMC&miR2disease
6	HOTAIR	Lnc2Cancer&LncRNADisease	hsa-mir-34a	dbDEMC&miR2disease
7	PVT1	Lnc2Cancer&LncRNADisease	hsa-mir-16	dbDEMC
8	GAS5	Lnc2Cancer	hsa-mir-143	dbDEMC&miR2disease
9	KCNQ1OT1	Lnc2Cancer	hsa-mir-146a	dbDEMC
10	UCA1	Lnc2Cancer&LncRNADisease	hsa-mir-92a	Unconfirmed
11	TUG1	Lnc2Cancer&LncRNADisease	hsa-mir-15a	dbDEMC
12	XIST	Lnc2Cancer&LncRNADisease	hsa-mir-19a	dbDEMC&miR2disease
13	AFAP1-AS1	Lnc2Cancer	hsa-mir-106b	dbDEMC&miR2disease
14	HULC	Lnc2Cancer	hsa-mir-125b	dbDEMC
15	BANCR	Unknown	hsa-mir-29a	dbDEMC&miR2disease

**Table 9:** Case study of 10 diseases predicted associations.

Disease	CircRNA	Evidence
Papillary thyroid carcinoma	circPVT1/hsa_circ_0001821	PMID: 35,520,785
Esophageal squamous cell carcinoma	hsa_circRNA_100782/circHIPK3/hsa_circ_0000284	PMID: 35,443,871
Rheumatoid arthritis	hsa_circRNA_100782/circHIPK3/hsa_circ_0000284	PMID: 35,291,619
Colorectal cancer	circSMARCA5/hsa_circ_0001445	PMID: 34,948,079
Breast cancer	circGFRA1/hsa_circ_005239	PMID: 34,668,628
Prostate cancer	hsa_circRNA_100782/circHIPK3/hsa_circ_0000284	PMID: 34,142,340
Gastric cancer	Cir-ITCH/hsa_circ_0001141/hsa_circ_001763	PMID: 33,060,778
Clear cell renal cell carcinoma	hsa_circRNA_100782/circHIPK3/hsa_circ_0000284	PMID: 32,409,849
Oral squamous cell carcinoma	circRNA_100290/hsa_circ_0013339/hsa_circ_100290	PMID: 31,187,488
Pancreatic ductal adenocarcinoma	hsa_circ_0001649	PMID: 29,969,694

### Visualization of feature representations

**Fig. 5:**
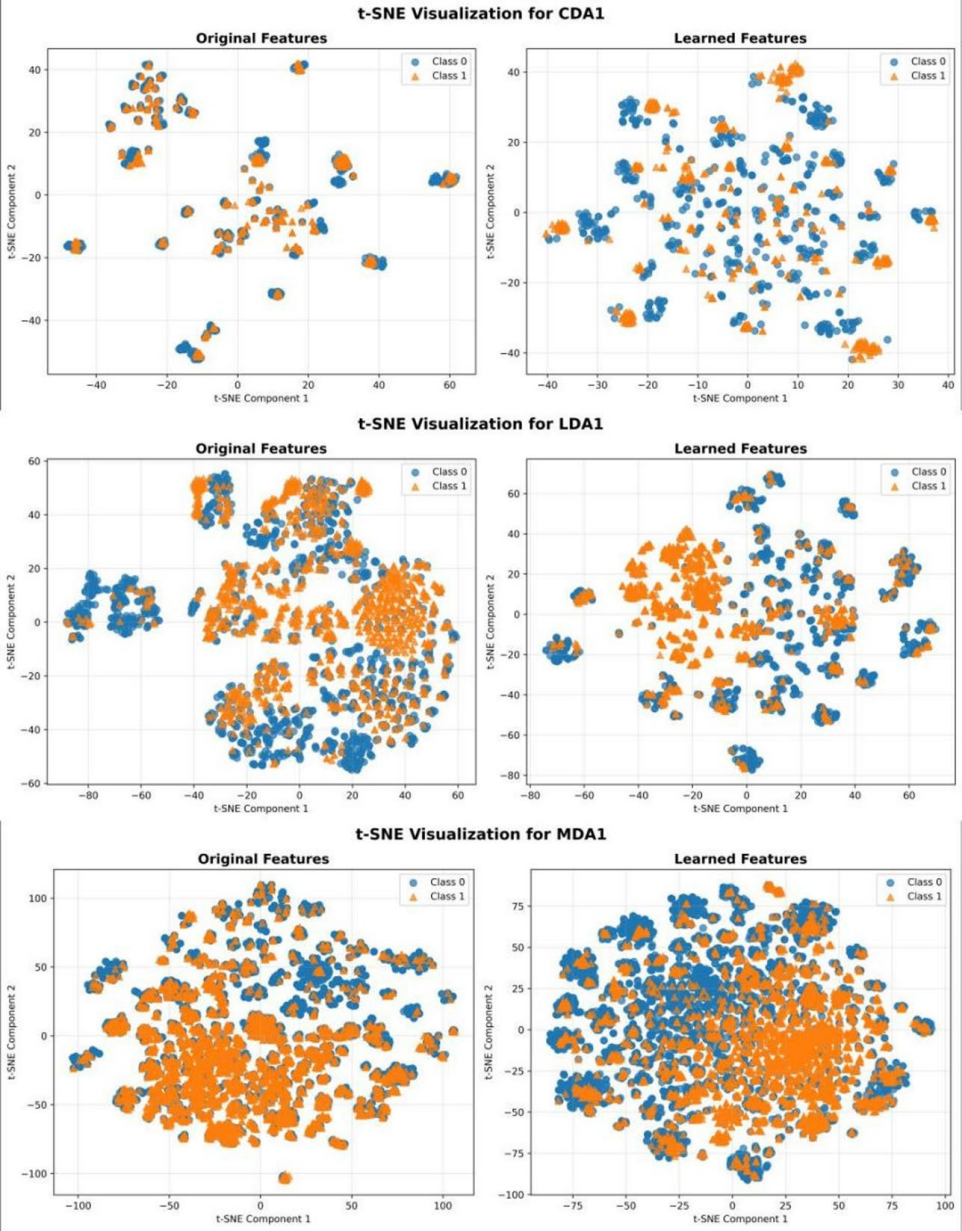
The t-SNE visualization of feature representations on CDA1, LDA1, and MDA1 datasets. The left column displays the distribution of original input features, while the right column shows the distribution of high-level node embeddings learned by the SSLGRDA model. Blue circles (Class 0) represent negative samples (non-associations), and orange triangles (Class 1) represent positive samples (known associations).

To intuitively evaluate the quality of the representations learned by SSLGRDA, we utilized t-SNE to visualize the feature distributions of ncRNA-disease pairs before and after model training. We selected three representative datasets (CDA1, LDA1, and MDA1) and randomly sampled balanced positive and negative pairs from the training sets. Figure 5 displays the visualization results.

As observed in the ‘Original Features’ plots, the positive and negative samples are heavily entangled with ambiguous boundaries, making them difficult to distinguish based on raw features alone. In contrast, in the ‘Learned Features’ space generated by SSLGRDA, the distribution of samples becomes more structured. The positive and negative classes form relatively concentrated regions, and the inter-class overlap is significantly reduced. This clearer separation demonstrates that SSLGRDA successfully captures discriminative topological and semantic patterns, projecting the data into a space that is much more favorable for the binary classification task.

## Discussion

We have successfully established a self-supervised learning model for the prediction of ncRNA-disease association. SSLGRDA offers a concise and generalizable model that boosts node embedding with self-supervised learning on graph. In our study, different contrastive strategies of graph self-supervised learning were considered, and contrasts topological structures and semantic features of the ncRNA-disease graph. Therefore, we can use a GNN module to capture more generalized network-level embedding and rely on a supervised label to perform predictions. We conduct comprehensive experiments and demonstrate significant improvements over competitive baselines on nine public datasets. Moreover, case studies on three RDA datasets demonstrate that our method achieves reliable prediction performance. In general, our model is highly flexible, applicable to both homogeneous and heterogeneous graphs, and can be easily extended to other applications such as microbe-disease association, microbe-drug association.

Although SSLGRDA performs well on RDA prediction, it still has a few limitations. First, failure to fully utilize ncRNA/disease similarity data, using only top-*k* similarities per ncRNA/disease, although reducing noisy information, may lose useful data. Second, lack of effective strategies to fuse node embeddings from different views together to form more robust features. In the future, we plan to address these limitations through two specific directions. First, to better utilize similarity data without introducing noise, we will explore graph attention mechanisms to assign learnable weights to similarity neighbors, replacing the rigid top-k thresholding. Second, to enhance feature integration, we intend to develop adaptive fusion modules or cross-view attention mechanisms that can dynamically weigh the importance of structural versus semantic views for each node, avoiding the limitations of simple concatenation.

## Supplementary Information

Below is the link to the electronic supplementary material.

Supplementary Material 1

Supplementary Material 2

Supplementary Material 3

Supplementary Material 4

Supplementary Material 5

Supplementary Material 6

Supplementary Material 7

## Data Availability

The source code and dataset are available at https://github.com/QingwWu/SSLGRDA
