# Supplementary material for: Self-supervised learning on graphs predicts non-coding RNA and disease associations
Source: Sci Rep. 2026 Jan 14;16:5231. doi: 10.1038/s41598-026-36030-2 (PMC12881540; doi:10.1038/s41598-026-36030-2)
Supplement: Supplementary file 1 — Supplementary Material 1 [file 41598_2026_36030_MOESM1_ESM.pdf]

Supplementary Table 1. Classification accuracy and ranking results of all methods on CDA2.

Dataset	Category	Model	AUC	AUPR	F1	Hits@10	Hits@50	Hits@100
CDA2	Contrastive	SSLG_GH_hete	0.70301	0.02426	0.04982	0.01379	0.04828	0.05517
	Contrastive	SSLG_GH_homo	0.86884	0.50849	0.14248	0.38974	0.48205	0.51538
	Contrastive	SSLG_GM_hete	0.69304	0.01631	0.00039	0.00345	0.02069	0.04207
	Contrastive	SSLG_GM_homo	**0.89502**	**0.56135**	0.13666	**0.47123**	**0.52308**	**0.55369**
	Generative	SSLG_MA_hete	0.68860	0.01456	0.03171	0.00690	0.01379	0.04138
	Generative	SSLG_MA_homo	0.77445	0.06332	0.06121	0.03231	0.07846	0.10769
	SSLG_Con	AFGRL	0.83797	0.21349	0.09398	0.12308	0.20000	0.24615
	SSLG_Gen	GAE	0.86076	0.38941	0.10486	0.26615	0.36615	0.42154
	RDAP	LR-GCN_hete	0.78509	0.03553	0.03099	0.02445	0.04672	0.06859
	RDAP	LR-GCN_homo	0.63128	0.02076	0.03992	0.00154	0.00462	0.00769
	RDAP	GMNN2CD	0.88083	0.37543	0.06667	0.28308	0.32154	0.36769
	RDAP	MINIMDA	0.63153	0.02597	0.03413	0.00308	0.00923	0.01692
	RDAP	MLGCN	0.89386	0.29819	0.12444	0.14897	0.25491	0.34398
	HeteGNN	GATNE	0.77824	0.39279	**0.20054**	0.28462	0.34615	0.43846
	HeteGNN	HGB	0.74834	0.11304	0.04569	0.05385	0.13846	0.23077
	HeteGNN	RGCN	0.69785	0.05528	0.09147	0.01538	0.05692	0.08769
**Dataset**	**Category**	**Model**	**MR↓**	**MRR**	**MR_L_R↓**	**MR_L_D↓**	**MRR_L_R**	**MRR_L_D**
CDA2	Contrastive	SSLG_GH_hete	3065.51	0.01028	8.88844	41.18202	0.27741	0.20208
	Contrastive	SSLG_GH_homo	1332.13	0.31949	3.54475	21.04291	0.69331	0.40285
	Contrastive	SSLG_GM_hete	3188.67	0.00331	9.03446	38.93422	0.25642	0.15140
	Contrastive	SSLG_GM_homo	**1077.69**	**0.39711**	3.25691	**17.12606**	**0.71732**	**0.44721**
	Generative	SSLG_MA_hete	3273.66	0.00245	8.52578	44.32874	0.24898	0.13049
	Generative	SSLG_MA_homo	2315.57	0.03306	4.88111	40.30515	0.49308	0.15235
	SSLG_Con	AFGRL	1646.60	0.08230	3.73967	27.72375	0.58772	0.38061
	SSLG_Gen	GAE	1414.63	0.14695	3.34045	25.07359	0.66769	0.37358
	RDAP	LR-GCN_hete	2179.15	0.00986	5.62245	43.11057	0.37585	0.14135
	RDAP	LR-GCN_homo	3742.93	0.00232	6.53740	59.09645	0.30184	0.03928
	RDAP	GMNN2CD	1222.84	0.20082	4.18378	22.27775	0.63170	0.25078
	RDAP	MINIMDA	3740.60	0.00237	5.78603	84.94015	0.39728	0.03900
	RDAP	MLGCN	1078.58	0.09570	**3.18575**	19.19458	0.69242	0.30902
	HeteGNN	GATNE	2274.72	0.21238	3.97903	30.38918	0.67128	0.38031
	HeteGNN	HGB	2581.28	0.01385	5.00161	38.34876	0.44651	0.09847
	HeteGNN	RGCN	3098.95	0.01278	6.03176	45.77498	0.41964	0.10251

↓ means the smaller the better. Best results in the experiment are highlighted in bold, and the second best result is underlined.
