# Supplementary material for: Self-supervised learning on graphs predicts non-coding RNA and disease associations
Source: Sci Rep. 2026 Jan 14;16:5231. doi: 10.1038/s41598-026-36030-2 (PMC12881540; doi:10.1038/s41598-026-36030-2)
Supplement: Supplementary file 2 — Supplementary Material 2 [file 41598_2026_36030_MOESM2_ESM.pdf]

Supplementary Table 4. Classification accuracy and ranking results of all methods on LDA3.

Dataset	Category	Model	AUC	AUPR	F1	Hits@10	Hits@50	Hits@100
LDA3	Contrastive	SSLG_GH_hete	0.90067	0.24994	0.26038	0.04522	0.22435	0.39130
	Contrastive	SSLG_GH_homo	**0.93725**	**0.46738**	**0.28476**	0.20058	0.45652	**0.58957**
	Contrastive	SSLG_GM_hete	0.90358	0.39946	0.26268	0.17739	0.36348	0.50435
	Contrastive	SSLG_GM_homo	0.93057	0.46217	0.27863	0.20895	0.43339	0.57304
	Generative	SSLG_MA_hete	0.89061	0.32503	0.24685	0.10748	0.30661	0.45983
	Generative	SSLG_MA_homo	0.90764	0.37689	0.22463	0.14087	0.38609	0.51478
	SSLG_Con	AFGRL	0.92161	0.43561	0.27982	0.20870	**0.46087**	0.53043
	SSLG_Gen	GAE	0.90748	0.39110	0.24990	0.14783	0.40522	0.53043
	RDAP	LR-GCN_hete	0.92051	0.32696	0.09101	0.13111	0.26798	0.40525
	RDAP	LR-GCN_homo	0.81430	0.19463	0.15797	0.05043	0.19652	0.29913
	RDAP	GMNN2CD	0.88922	0.40442	0.07467	**0.29391**	0.34087	0.37913
	RDAP	MINIMDA	0.80974	0.20427	0.13740	0.10435	0.21304	0.27826
	RDAP	MLGCN	0.92717	0.42352	0.28688	0.17052	0.39343	0.52676
	HeteGNN	GATNE	0.81529	0.35405	0.21477	0.02295	0.14754	0.29508
	HeteGNN	HGB	0.79259	0.17150	0.14211	0.04348	0.19130	0.25217
	HeteGNN	RGCN	0.80925	0.14507	0.17603	0.02087	0.12174	0.19478

Dataset	Category	Model	MR↓	MRR	MR_L_R↓	MR_L_D↓	MRR_L_R	MRR_L_D
LDA3	Contrastive	SSLG_GH_hete	479.08	0.02611	5.64467	6.85807	0.48809	0.50936
	Contrastive	SSLG_GH_homo	**305.47**	0.09910	**3.92091**	**4.40599**	**0.67536**	**0.56529**
	Contrastive	SSLG_GM_hete	473.26	0.09585	6.01584	6.81801	0.45665	0.52432
	Contrastive	SSLG_GM_homo	337.90	0.10934	4.20909	4.75334	0.66045	0.55992
	Generative	SSLG_MA_hete	532.23	0.07207	5.89392	6.84461	0.45912	0.49492
	Generative	SSLG_MA_homo	448.86	0.10578	5.34718	5.45332	0.57110	0.50014
	SSLG_Con	AFGRL	374.16	0.07757	4.44631	4.94479	0.63681	0.48316
	SSLG_Gen	GAE	441.26	0.07397	5.15677	5.35872	0.60025	0.51718
	RDAP	LR-GCN_hete	379.21	0.08539	5.13553	6.65044	0.49988	0.55015
	RDAP	LR-GCN_homo	884.59	0.03264	6.86036	10.90522	0.43110	0.35416
	RDAP	GMNN2CD	538.37	**0.13991**	5.66668	7.57449	0.57492	0.37130
	RDAP	MINIMDA	906.26	0.04337	6.11128	10.27319	0.46623	0.39140
	RDAP	MLGCN	354.23	0.08084	4.51732	4.88069	0.61427	0.53263
	HeteGNN	GATNE	569.16	0.01518	6.65293	8.32091	0.40549	0.30362
	HeteGNN	HGB	1007.17	0.03132	6.16173	14.54444	0.49555	0.34584
	HeteGNN	RGCN	926.35	0.01646	7.09869	10.79109	0.45843	0.32921

↓ means the smaller the better. Best results in the experiment are highlighted in bold, and the second best result is underlined.
