# Supplementary material for: Self-supervised learning on graphs predicts non-coding RNA and disease associations
Source: Sci Rep. 2026 Jan 14;16:5231. doi: 10.1038/s41598-026-36030-2 (PMC12881540; doi:10.1038/s41598-026-36030-2)
Supplement: Supplementary file 3 — Supplementary Material 3 [file 41598_2026_36030_MOESM3_ESM.pdf]

Supplementary Table 2. Classification accuracy and ranking results of all methods on CDA3.

Dataset	Category	Model	AUC	AUPR	F1	Hits@10	Hits@50	Hits@100
CDA3	Contrastive	SSLG_GH_hete	0.72396	0.02023	0.03226	0.00661	0.02322	0.05645
	Contrastive	SSLG_GH_homo	**0.81889**	**0.19141**	**0.09112**	0.10420	**0.19328**	**0.26387**
	Contrastive	SSLG_GM_hete	0.75748	0.02282	0.04915	0.00328	0.01317	0.05262
	Contrastive	SSLG_GM_homo	0.80200	0.14740	0.07210	0.07412	0.13983	0.19748
	Generative	SSLG_MA_hete	0.63356	0.02712	0.02724	0.01639	0.04596	0.09213
	Generative	SSLG_MA_homo	0.75259	0.04489	0.05311	0.08403	0.08739	0.09916
	SSLG_Con	AFGRL	0.80744	0.11368	0.07602	0.04202	0.15126	0.18487
	SSLG_Gen	GAE	0.79100	0.15853	0.06872	0.07563	0.15966	0.23866
	RDAP	LR-GCN_hete	0.76560	0.03816	0.02828	0.01676	0.05931	0.09508
	RDAP	LR-GCN_homo	0.59592	0.01794	0.03995	0.00168	0.00168	0.00168
	RDAP	GMNN2CD	0.77868	0.18692	0.01613	**0.11765**	0.12437	0.23025
	RDAP	MINIMDA	0.62977	0.02940	0.04568	0.00840	0.02017	0.02689
	RDAP	MLGCN	0.79148	0.11158	0.08772	0.04874	0.13445	0.17815
	HeteGNN	GATNE	0.59631	0.07975	0.07869	0.04202	0.09244	0.14286
	HeteGNN	HGB	0.67266	0.04837	0.04156	0.00840	0.09244	0.11765
	HeteGNN	RGCN	0.57907	0.02306	0.04373	0.00504	0.02185	0.04370

Dataset	Category	Model	MR↓	MRR	MR_L_R↓	MR_L_D↓	MRR_L_R	MRR_L_D
CDA3	Contrastive	SSLG_GH_hete	2600.90	0.00431	9.19383	35.54084	0.25374	0.14254
	Contrastive	SSLG_GH_homo	**1617.71**	0.06816	4.18378	30.95399	**0.57592**	0.28592
	Contrastive	SSLG_GM_hete	2273.31	0.00283	6.46822	41.82888	0.37263	0.09304
	Contrastive	SSLG_GM_homo	1856.07	0.04363	4.41475	33.16684	0.54066	**0.29302**
	Generative	SSLG_MA_hete	3434.31	0.00816	7.63728	53.61047	0.29291	0.07576
	Generative	SSLG_MA_homo	2319.05	**0.08529**	5.24326	37.69896	0.45562	0.14203
	SSLG_Con	AFGRL	1786.77	0.01622	**3.98718**	38.09543	0.56806	0.24865
	SSLG_Gen	GAE	1939.49	0.05147	4.56504	32.72077	0.53636	0.28393
	RDAP	LR-GCN_hete	2171.80	0.01170	6.38680	35.30304	0.37793	0.11499
	RDAP	LR-GCN_homo	3747.50	0.00090	7.74308	52.44011	0.27625	0.04970
	RDAP	GMNN2CD	2074.33	0.08482	5.28838	32.96694	0.50715	0.22440
	RDAP	MINIMDA	3434.12	0.00720	5.85386	77.18514	0.38578	0.05320
	RDAP	MLGCN	1935.29	0.03002	4.56757	**28.45889**	0.51975	0.25047
	HeteGNN	GATNE	3782.73	0.03388	7.67568	46.01163	0.36848	0.23352
	HeteGNN	HGB	3067.54	0.00625	5.98048	51.49867	0.42456	0.04962
	HeteGNN	RGCN	3944.31	0.00393	8.02187	49.56606	0.29801	0.07116

↓ means the smaller the better. Best results in the experiment are highlighted in bold, and the second best result is underlined.
