# Supplementary material for: Self-supervised learning on graphs predicts non-coding RNA and disease associations
Source: Sci Rep. 2026 Jan 14;16:5231. doi: 10.1038/s41598-026-36030-2 (PMC12881540; doi:10.1038/s41598-026-36030-2)
Supplement: Supplementary file 4 — Supplementary Material 4 [file 41598_2026_36030_MOESM4_ESM.pdf]

Supplementary Table 3. Classification accuracy and ranking results of all methods on LDA2.

Dataset	Category	Model	AUC	AUPR	F1	Hits@10	Hits@50	Hits@100
LDA2	Contrastive	SSLG_GH_hete	0.96328	0.87411	0.73092	0.64689	0.79202	0.83991
	Contrastive	SSLG_GH_homo	0.95926	0.85220	0.68596	0.59016	0.74645	0.80328
	Contrastive	SSLG_GM_hete	0.96243	0.87139	0.72252	0.64616	0.78531	0.83915
	Contrastive	SSLG_GM_homo	0.96157	0.86679	0.67883	**0.65475**	0.76531	0.81554
	Generative	SSLG_MA_hete	**0.96429**	**0.87413**	**0.74030**	0.61410	**0.79908**	**0.84990**
	Generative	SSLG_MA_homo	0.95836	0.83466	0.63557	0.54426	0.71869	0.79541
	SSLG_Con	AFGRL	0.95959	0.84707	0.67183	0.62295	0.73115	0.78033
	SSLG_Gen	GAE	0.95405	0.83530	0.66707	0.60852	0.72984	0.78820
	RDAP	LR-GCN_hete	0.96158	0.82286	0.47769	0.41670	0.70936	0.79215
	RDAP	LR-GCN_homo	0.90077	0.73985	0.57463	0.41639	0.60197	0.68787
	RDAP	GMNN2CD	0.94565	0.83014	0.13106	0.52852	0.73705	0.79279
	RDAP	MINIMDA	0.90431	0.72507	0.62372	0.28197	0.54754	0.73115
	RDAP	MLGCN	0.95272	0.82966	0.65663	0.60036	0.73380	0.78024
	HeteGNN	GATNE	0.81921	0.43318	0.25781	0.07213	0.26557	0.40984
	HeteGNN	HGB	0.93157	0.70491	0.59560	0.24918	0.47213	0.68197
	HeteGNN	RGCN	0.91674	0.63982	0.29725	0.02689	0.13443	0.26426
**Dataset**	**Category**	**Model**	**MR↓**	**MRR**	**MR_L_R↓**	**MR_L_D↓**	**MRR_L_R**	**MRR_L_D**
LDA2	Contrastive	SSLG_GH_hete	113.35	0.39019	3.48103	4.32013	0.62360	0.52151
	Contrastive	SSLG_GH_homo	116.16	0.37689	3.87390	3.34156	0.63059	0.63625
	Contrastive	SSLG_GM_hete	118.36	0.37944	3.48812	4.42744	0.61645	0.51411
	Contrastive	SSLG_GM_homo	119.25	**0.44800**	3.99418	3.49063	0.62460	0.60468
	Generative	SSLG_MA_hete	110.95	0.36497	3.48422	4.24085	0.62244	0.52968
	Generative	SSLG_MA_homo	128.64	0.44346	3.74683	3.42218	**0.67905**	0.63725
	SSLG_Con	AFGRL	115.38	0.33706	4.38427	**2.93205**	0.58765	**0.65899**
	SSLG_Gen	GAE	130.68	0.37251	4.05949	3.43246	0.64674	0.62659
	RDAP	LR-GCN_hete	**109.77**	0.26192	**3.26043**	4.21899	0.62861	0.51356
	RDAP	LR-GCN_homo	281.94	0.24761	5.50595	5.58965	0.58310	0.44743
	RDAP	GMNN2CD	168.17	0.28729	4.67280	4.08172	0.62104	0.53265
	RDAP	MINIMDA	271.90	0.13940	4.38936	4.23737	0.63112	0.56297
	RDAP	MLGCN	149.68	0.42324	4.09388	3.72924	0.65560	0.57665
	HeteGNN	GATNE	557.10	0.04204	6.29636	8.65770	0.45416	0.27282
	HeteGNN	HGB	211.50	0.14442	4.30708	4.92540	0.60597	0.50019
	HeteGNN	RGCN	257.12	0.06620	5.09058	5.09830	0.55718	0.44708

↓ means the smaller the better. Best results in the experiment are highlighted in bold, and the second best result is underlined.
