# Supplementary material for: Self-supervised learning on graphs predicts non-coding RNA and disease associations
Source: Sci Rep. 2026 Jan 14;16:5231. doi: 10.1038/s41598-026-36030-2 (PMC12881540; doi:10.1038/s41598-026-36030-2)
Supplement: Supplementary file 5 — Supplementary Material 5 [file 41598_2026_36030_MOESM5_ESM.pdf]

Supplementary Table 6. Classification accuracy and ranking results of all methods on MDA3.

Dataset	Category	Model	AUC	AUPR	F1	Hits@10	Hits@50	Hits@100
MDA3	Contrastive	SSLG_GH_hete	0.94769	0.52681	0.33661	0.05209	0.12248	0.19554
	Contrastive	SSLG_GH_homo	<u>0.95031</u>	<u>0.55473</u>	<u>0.33439</u>	0.07306	0.16620	0.21881
	Contrastive	SSLG_GM_hete	0.94169	0.51859	0.28269	0.08433	0.16598	0.22164
	Contrastive	SSLG_GM_homo	**0.95302**	**0.57457**	**0.33830**	0.05577	0.15839	0.23503
	Generative	SSLG_MA_hete	0.94375	0.48854	0.28889	0.04986	0.12861	0.18595
	Generative	SSLG_MA_homo	0.93236	0.45049	0.27298	**0.25923**	**0.25923**	<u>0.25923</u>
	SSLG_Con	AFGRL	0.94800	0.52007	0.32932	0.04852	0.11768	0.18349
	SSLG_Gen	GAE	0.94361	0.50827	0.31427	0.07719	0.13720	0.19163
	RDAP	LR-GCN_hete	0.94130	0.46874	0.22508	0.05405	0.11355	0.16454
	RDAP	LR-GCN_homo	0.88066	0.31858	0.22389	0.01372	0.05142	0.08723
	RDAP	GMNN2CD	0.87248	0.39510	0.06962	<u>0.13530</u>	<u>0.23335</u>	**0.28678**
	RDAP	MINIMDA	0.93672	0.52576	0.31337	0.08221	0.17903	0.23257
	RDAP	MLGCN	0.94539	0.51161	0.32966	0.08107	0.13147	0.19927
	HeteGNN	GATNE	0.65066	0.04366	0.08597	0.00015	0.00112	0.00279
	HeteGNN	HGB	0.89321	0.25054	0.20793	0.00558	0.02733	0.04741
	HeteGNN	RGCN	0.82046	0.16201	0.07586	0.00190	0.01015	0.02019

Dataset	Category	Model	MR↓	MRR	MR_L_R↓	MR_L_D↓	MRR_L_R	MRR_L_D
MDA3	Contrastive	SSLG_GH_hete	2938.29	0.02793	8.79363	20.12900	0.41378	0.25261
	Contrastive	SSLG_GH_homo	<u>2770.71</u>	0.07891	7.89596	19.05475	**0.48720**	<u>0.26509</u>
	Contrastive	SSLG_GM_hete	3335.02	0.03963	8.91289	20.86827	0.41167	0.24243
	Contrastive	SSLG_GM_homo	**2618.60**	0.03018	<u>7.81529</u>	**17.85344**	<u>0.48120</u>	**0.27420**
	Generative	SSLG_MA_hete	3217.33	0.02811	8.91087	21.17981	0.41394	0.21806
	Generative	SSLG_MA_homo	3846.36	**0.25998**	10.33165	24.65186	0.43649	0.24091
	SSLG_Con	AFGRL	2898.76	0.02841	**7.62851**	<u>18.54560</u>	0.47934	0.23606
	SSLG_Gen	GAE	3142.99	0.08124	8.76138	21.11195	0.45479	0.23654
	RDAP	LR-GCN_hete	3271.49	0.03033	8.29679	20.93848	0.43211	0.21643
	RDAP	LR-GCN_homo	6652.04	0.00982	9.81913	41.21820	0.43095	0.15604
	RDAP	GMNN2CD	7288.43	0.07333	9.85073	42.55038	0.46093	0.07947
	RDAP	MINIMDA	3526.71	0.04398	8.69149	21.28287	0.43410	0.23281
	RDAP	MLGCN	3123.55	<u>0.08483</u>	8.51834	19.82057	0.44156	0.25124
	HeteGNN	GATNE	19964.84	0.00023	32.88510	48.27760	0.08526	0.07298
	HeteGNN	HGB	6103.78	0.00366	9.29969	28.86185	0.42693	0.15797
	HeteGNN	RGCN	10261.63	0.00225	16.49847	46.50452	0.25010	0.09950

↓ means the smaller the better. Best results in the experiment are highlighted in bold, and the second best result is underlined.
