# Supplementary material for: Self-supervised learning on graphs predicts non-coding RNA and disease associations
Source: Sci Rep. 2026 Jan 14;16:5231. doi: 10.1038/s41598-026-36030-2 (PMC12881540; doi:10.1038/s41598-026-36030-2)
Supplement: Supplementary file 6 — Supplementary Material 6 [file 41598_2026_36030_MOESM6_ESM.pdf]

Supplementary Table 5. Classification accuracy and ranking results of all methods on MDA2.

Dataset	Category	Model	AUC	AUPR	F1	Hits@10	Hits@50	Hits@100
MDA2	Contrastive	SSLG_GH_hete	0.93883	0.46842	0.30549	0.04309	0.13444	0.20773
	Contrastive	SSLG_GH_homo	0.94171	0.47398	0.30430	0.03959	0.12707	0.21179
	Contrastive	SSLG_GM_hete	0.93399	0.46367	0.30804	0.05009	0.15543	0.21584
	Contrastive	SSLG_GM_homo	**0.94354**	**0.52196**	**0.31448**	0.05764	0.17617	0.27326
	Generative	SSLG_MA_hete	0.93234	0.45359	0.30106	0.04420	0.12081	0.19521
	Generative	SSLG_MA_homo	0.91820	0.36584	0.22848	**0.18840**	0.18840	0.19871
	SSLG_Con	AFGRL	0.93133	0.46786	0.29864	0.03407	0.13444	0.20810
	SSLG_Gen	GAE	0.92937	0.43312	0.27815	0.04365	0.10902	0.19540
	RDAP	LR-GCN_hete	0.92004	0.38672	0.20767	0.03597	0.10434	0.16088
	RDAP	LR-GCN_homo	0.86835	0.32884	0.20920	0.03186	0.09779	0.14180
	RDAP	GMNN2CD	0.91698	0.52187	0.06231	0.09679	**0.25595**	**0.32628**
	RDAP	MINIMDA	0.91407	0.41280	0.19476	0.04899	0.12891	0.18729
	RDAP	MLGCN	0.93534	0.45076	0.29626	0.05433	0.12855	0.20144
	HeteGNN	GATNE	0.65028	0.04391	0.08569	0.00025	0.00092	0.00184
	HeteGNN	HGB	0.88375	0.18232	0.18624	0.00368	0.01842	0.03039
	HeteGNN	RGCN	0.80725	0.15554	0.06185	0.00331	0.01602	0.03057

Dataset	Category	Model	MR↓	MRR	MR_L_R↓	MR_L_D↓	MRR_L_R	MRR_L_D
MDA2	Contrastive	SSLG_GH_hete	2199.96	0.02537	8.36475	15.94755	0.40922	0.31673
	Contrastive	SSLG_GH_homo	2097.37	0.02837	8.45766	13.20929	**0.49954**	0.34103
	Contrastive	SSLG_GM_hete	2437.27	0.02616	9.07621	15.80073	0.39883	0.31493
	Contrastive	SSLG_GM_homo	**2081.44**	0.03046	**8.04499**	**13.00662**	0.48659	**0.35407**
	Generative	SSLG_MA_hete	2502.58	0.03199	9.13308	16.42646	0.40734	0.31554
	Generative	SSLG_MA_homo	3012.08	**0.18958**	10.91122	17.78006	0.41518	0.24375
	SSLG_Con	AFGRL	2470.17	0.01873	8.89780	14.20784	0.47003	0.31993
	SSLG_Gen	GAE	2541.36	0.04884	9.45963	15.48117	0.43544	0.28813
	RDAP	LR-GCN_hete	2876.55	0.01668	9.68723	17.98385	0.36198	0.21618
	RDAP	LR-GCN_homo	4735.15	0.01886	12.33773	26.75262	0.36963	0.19125
	RDAP	GMNN2CD	2789.73	0.04100	8.90800	20.52226	0.49290	0.23450
	RDAP	MINIMDA	3091.24	0.02844	9.62945	16.49699	0.40315	0.24539
	RDAP	MLGCN	2383.77	0.05697	8.82668	14.77133	0.45628	0.31983
	HeteGNN	GATNE	12881.46	0.00028	30.77238	32.06155	0.08890	0.11551
	HeteGNN	HGB	4282.46	0.00292	9.64871	20.76208	0.36311	0.23532
	HeteGNN	RGCN	7100.25	0.00311	17.34755	32.95693	0.25407	0.13561

↓ means the smaller the better. Best results in the experiment are highlighted in bold, and the second best result is underlined.
