# Supplementary material for: Self-supervised learning on graphs predicts non-coding RNA and disease associations
Source: Sci Rep. 2026 Jan 14;16:5231. doi: 10.1038/s41598-026-36030-2 (PMC12881540; doi:10.1038/s41598-026-36030-2)
Supplement: Supplementary file 7 — Supplementary Material 7 [file 41598_2026_36030_MOESM7_ESM.pdf]

Supplementary Table 7. Statistical significance test (Paired t-test) on all ncRNA-disease datasets.

Dataset	Competing method	Metric	P-values
CDA1	GAE	AUC	1.24e-02
		AUPR	8.56e-03
CDA2	GATNE	AUC	3.12e-02
		AUPR	4.45e-02
CDA3	GMNN2CD	AUC	2.10e-03
		AUPR	1.89e-03
LDA1	MLGCN	AUC	6.78e-04
		AUPR	9.21e-04
LDA2	AFGRL	AUC	1.05e-02
		AUPR	2.34e-02
LDA3	AFGRL	AUC	8.76e-04
		AUPR	5.43e-04
MDA1	AFGRL	AUC	1.12e-02
		AUPR	9.87e-03
MDA2	GMNN2CD	AUC	5.67e-03
		AUPR	7.23e-03
MDA3	MINIMDA	AUC	2.31e-02
		AUPR	3.05e-02

Note: Comparison between SSLGRDA (best variant) and the best-performing baseline method.
